# Monitoring of SARS-CoV-2 antibodies using dried blood spot for at-home collection

**DOI:** 10.1038/s41598-022-09699-4

**Published:** 2022-04-06

**Authors:** Peyton K. Miesse, Bradley B. Collier, Russell P. Grant

**Affiliations:** Center for Esoteric Testing, Laboratory Corporation of America Holdings, Burlington, NC USA

**Keywords:** Biomarkers, Assay systems

## Abstract

The utilization of vaccines to fight the spread of SARS-CoV-2 has led to a growing need for expansive serological testing. To address this, an EUA approved immunoassay for detection of antibodies to SARS-CoV-2 in venous serum samples was investigated for use with dried blood spot (DBS) samples. Results from self-collected DBS samples demonstrated a 98.1% categorical agreement to venous serum with a correlation (R) of 0.9600 while professionally collected DBS samples demonstrated a categorical agreement of 100.0% with a correlation of 0.9888 to venous serum. Additional studies were performed to stress different aspects of at-home DBS collection, including shipping stability, effects of interferences, and other sample-specific robustness studies. These studies demonstrated a categorical agreement of at least 95.0% and a mean bias less than ± 20.0%. Furthermore, the ability to track antibody levels following vaccination with the BioNTech/Pfizer vaccine was demonstrated with serial self-collected DBS samples from pre-dose (Day 0) out to 19 weeks.

## Introduction

Despite the implementation of several measures to slow the spread of the disease, severe acute respiratory syndrome coronavirus 2 (SARS-CoV-2) continues to be an international public health emergency due to rapid human-to-human transmission and prevalence of asymptomatic carriers^1,2^. Initially, many countries implemented physical distancing protocols and/or lockdown restrictions^3^. In addition, diagnostic tests were quickly developed and granted FDA emergency use authorization (EUA) in order to identify individuals with active SARS-CoV-2 infections^4^. Although social distancing and diagnostic testing continue to be vital to ending the pandemic, the advent of SARS-CoV-2 vaccines provides a more robust means of limiting the spread of the virus^4^. By triggering the body’s natural immune response, vaccines initiate the creation of antibodies that can neutralize the virus upon infection and ultimately reduce the severity of infections as well as transmission of the virus^5^. Unfortunately, the lifespan of circulating SARS-CoV-2 antibodies and the requisite titer to yield protective immunity against SARS-CoV-2 is still unclear^5,6^. These concerns are further confounded by potential immunological differences between immunization versus native infection and the evolutionary nature of the virus (i.e. virus variants)^5^.

For these reasons, serological testing or measurement of circulating antibodies is becoming increasingly important in order to determine if an individual has generated antibodies to SARS-CoV-2 as a result of infection and/or vaccination^4,7^. Serological measurements are typically performed using serum or plasma samples obtained intravenously, however, dried blood spot (DBS) samples offer an alternative means of sample collection with several advantages over traditional phlebotomy. In addition to utilizing a smaller volume of blood (~ 50 µL of blood/spot) and less stringent shipping requirements, DBS cards can be successfully collected in the home setting with minimal training^8–10^. In addition, this alternate sample type has demonstrated utility for the detection of a variety of viral pathogens^9,11–14^.

**Figure 1:**
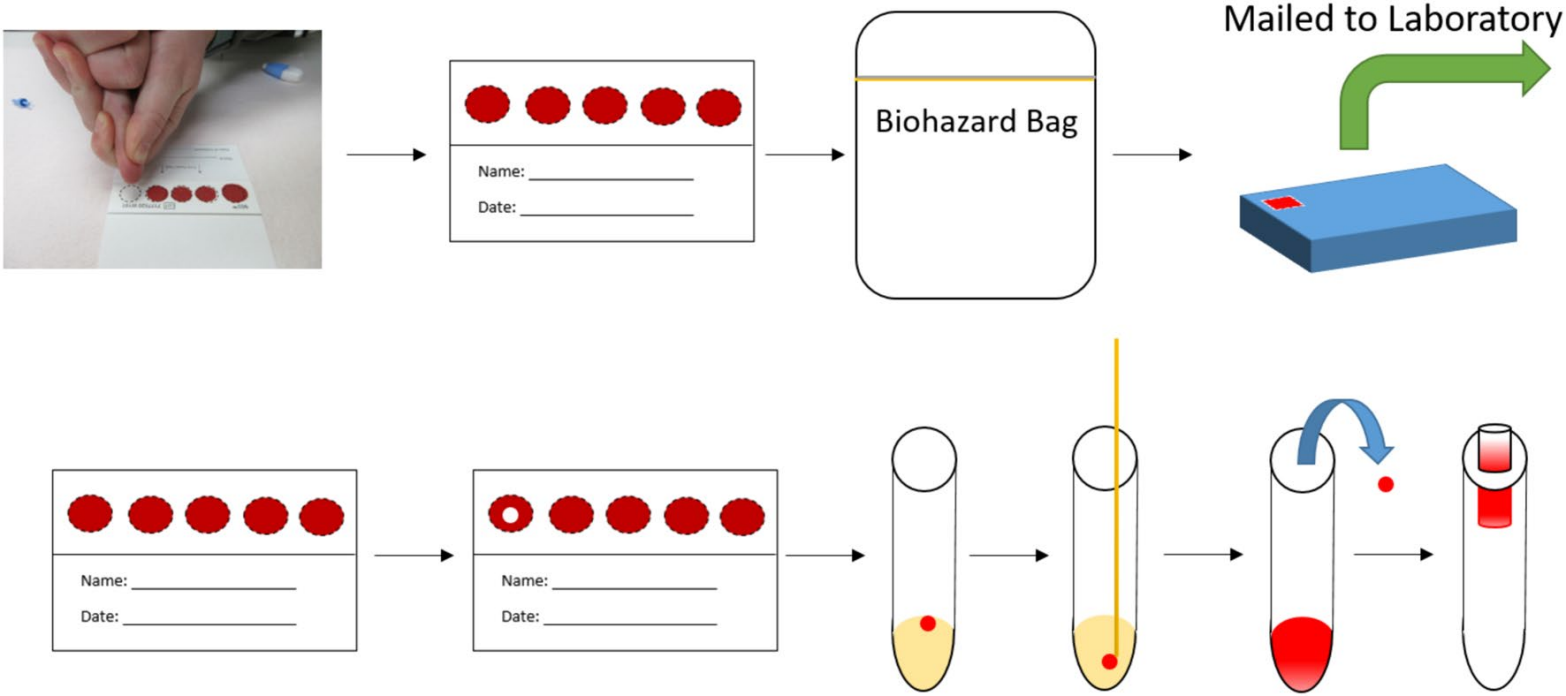
General process by which a patient would self-collect a DBS sample at home, send it into the laboratory, and how it is extracted and measured in the laboratory.

Since the start of the pandemic, many laboratories and researchers have investigated the use of DBS collection for both qualitative and quantitative detection of SARS-CoV-2 antibodies^10,15–21^. These assays have demonstrated high sensitivity and specificity when comparing DBS results to plasma or serum in proof-of-concept studies (Table 1). However, further investigation including studies based on regulatory guidance is required prior to utilization of DBS samples for at-home self-collection^22^. In the work presented here, we demonstrate the ability to measure SARS-CoV-2 antibodies from DBS samples using the Roche Elecsys Anti-SARS-CoV-2 S electrochemiluminescence immunoassay which has received emergency use authorization (EUA) for semi-quantitative measurement of antibodies in venous serum and plasma samples^23^. The feasibility of using this assay to measure SARS-CoV-2 antibodies has been demonstrated previously^16^. However, the studies performed herein represent a more rigorous testing of this assay including a simplified extraction process, a reduction in the assay’s reporting limit for DBS samples, and demonstration of sample self-collection. An example of the proposed sample and assay work flow can be found in Fig. 1. Lastly, serial monitoring of SARS-CoV-2 antibodies was demonstrated using self-collected DBS samples. FDA EUA guidance for serological testing for at-home sample collection was utilized where applicable^22^.

**Table 1:** Comparison of different DBS studies performed for measurement of SARS-CoV-2 antibodies.

Assay (instrument)	Samples	Comparator method	NPA, PPA, and TA^‡^	Additional studies performed	References
Semi-quantitative^a^ (autoanalyzer)	33 pos. 78 neg.	Diagnostic RT-PCR	NPA: 100.0% PPA: 97.0% TA: 99.1%	Sensitivity, robustness, imprecision, stability, interferences, and other studies	This work
Semi-quantitative^a^ (autoanalyzer)	34 pos.^†^ 75 neg.^†^	Diagnostic RT-PCR	NPA: 97.3% PPA: 97.1% TA: 97.2%	Sensitivity, robustness, imprecision, stability, interferences, and other studies	This work
Semi-quantitative^a^ (autoanalyzer)	52 pos. 11 neg.	Diagnostic RT-PCR	NPA: 88.5% PPA: 100% TA: 90.5%	None	^16^
Qualitative^b^ (autoanalyzer)	18 pos. 177 neg.	Venous plasma antibody measurements	NPA: 88.8%% PPA: 100% TA: 99.0%	Sample quality assessment only	^15^
Qualitative^b^ (autoanalyzer)	373 pos. 1337 neg.	Venous plasma antibody measurements	NPA: 99.2% PPA: 98.7% TA: 98.8%	None	^20^
Qualitative^c^ (plate reader)	108 pos. 281 neg.*	Venous serum antibody measurements	NPA: 98.1% PPA: 98.6% TA: 98.5%	Sample quality assessment only	^19^
Qualitative^d^ (plate reader)	111 pos. 278 neg.*	Venous serum antibody measurements	NPA: 94.7% PPA: 98.9% TA: 97.7%	Sample quality assessment only	^19^
Qualitative^c^ (plate reader)	35 pos.^†^ 30 neg.^†^	Diagnostic RT-PCR	NPA: 82.8% PPA: 76.7% TA: 80.0%	Stability, robustness, imprecision, and other studies	^17^
Qualitative^c^ (plate reader)	22 pos. 21 neg.	Diagnostic RT-PCR	NPA: 90.9% PPA: 100% TA: 95.3%	Stability, robustness, imprecision, and other studies	^10^
Qualitative^e^ (plate reader)	22 pos. 21 neg.	Diagnostic RT-PCR	NPA: 86.4% PPA: 95.2% TA: 90.7%	Stability, robustness, imprecision, and other studies	^10^
Qualitative^f^ (flow cytometer)	51 pos. 108 neg.	Venous serum antibody measurements	NPA: 98.0% PPA: 100% TA: 99.4%	Imprecision	^18^

*Borderline measurements were treated as negative for ease of method comparison.

^†^DBS samples were self-collected by the patient.

^‡^Total agreement.

^a^Roche Elecsys Anti-SARS-CoV-2 S immunoassay.

^b^Roche Elecsys anti-SARS-CoV-2 immunoassay.

^c^Euroimmun anti-SARS-CoV-2 IgG ELISA.

^d^Euroimmun anti-SARS-CoV-2 NCP IgG ELISA.

^e^Epitope Diagnostics Novel Coronavirus COVID-19 IgG ELISA kit.

^f^Luminex xMAP SARS-CoV-2 multi-antigen assay.

## Material and Methods

### Sample extraction and measurement

For extraction of DBS samples, two ¼” diameter round hole punches were taken from regions of the DBS card that were saturated with blood (i.e. no white is visible on the punches) and placed into a single 16 × 75 mm polypropylene tube. Punches were then submerged in 150 µl of Roche Universal Diluent (07299001190) using a wooden applicator. Tubes were then placed on a micro plate shaker (VWR, 12620-926) at 240 rpm for one hour at room temperature (20–25 °C). Following extraction, remnant solution was squeezed out of the punches which were then discarded. The remaining extract (~ 100 µL) was then transferred into a Hitachi microcup (system dead volume of 50 µL) for measurement on a Roche Cobas 8000 e801 immunoassay module.

Measurement of SARS-CoV-2 antibodies was performed using the Roche Elecsys Anti-SARS-CoV-2 S assay which has received EUA approval for the semi-quantitative measurement of total SARS-CoV-2 antibodies in serum and plasma samples. With the assistance of Roche, the lower numerical reporting limit was reduced from 0.400 U/mL (venous sample limit of quantitation) to 0.000 U/mL. This is necessary as samples obtained using DBS are diluted (~ tenfold) through the extraction process. By reducing the reporting limit, a lower LOQ for DBS extracts could be investigated and reduce the possibility of false negative results for patients with serum antibody results just above the serum clinical cutoff (0.800 U/mL)^23^. For example, a patient with serum antibody results of 3.00 U/mL would have a DBS result of 0.300 U/mL (assuming tenfold dilution and complete analyte recovery) which is less than the EUA approved assay LOQ (0.400 U/mL). No other EUA assay parameters were modified.

### Clinical agreement studies

Paired venous serum and DBS samples were collected from individuals with previous COVID-19 infection (based on EUA approved RT-PCR diagnostic assays, n = 36) as well as from presumed negative individuals (n = 84). Donors previously infected with COVID-19 had nasopharyngeal samples collected and tested (using EUA approved methodologies for detection of SARS-CoV-2) between October and December 2020. All DBS and serum samples for this study were collected in January 2021.

Venous samples were obtained using traditional venipuncture techniques while DBS samples were obtained following sterilization of the fingertip with an alcohol pad and lancing the finger with a high flow contact-activated lancet (BD #366594). After wiping the first drop of blood with a gauze pad, blood was applied to the DBS card (Eastern Business Forms 903™ Dried Blood Spot Card, 10550021) to fill all five spots (~ 50 μL/spot). No instructions were provided regarding milking or squeezing of the finger after the fingerstick. Following collection, DBS samples were dried for three hours at room temperature (20–25 °C) and then placed into a plastic specimen pouch (without desiccant) for storage until testing. For self-collection of DBS samples, all donors provided samples in a home-like setting using detailed instructions for use to assist with the process described above. To rule out potential active asymptomatic infection for presumed negative donors, a self-collected nasal swab was procured concurrently and analyzed using Labcorp’s EUA approved RT-PCR assay for SARS-COV-2^24^.

Participants within this study represented individuals with varying levels of education and self-collection experience. All studies were performed in accordance with relevant guidelines and regulations under two Institutional Review Board (IRB) approved protocols: SQNM-RND-103 (IRB number 520100174, study number 1269845) and SCMM-RND-402 (IRB number 520180046, study number 1270479). These protocols were reviewed and approved by WCG IRB. Informed consent was obtained from all subjects.

### Creation of contrived blood DBS samples

For specific validation studies, contrived blood samples were created due to the difficulty of obtaining DBS samples at specific concentrations and in large volumes. These samples were generated by mixing venous serum (screened for SARS-CoV-2 antibodies) with packed red blood cells to create samples with 40% hematocrit. Red blood cells were obtained intravenously from a seronegative donor with Type O blood using EDTA tubes. After mixing, the contrived blood samples were pipetted onto DBS cards (~ 50 μL/spot) which were then allowed to dry for 3 h at room temperature prior to storage. For many studies (specifically those regarding sample and assay robustness), contrived blood samples were created such that approximately 25% of the samples utilized were negative samples within 5× the DBS assay cutoff, 50% were positive samples within 5× the cutoff, and 25% were greater than 5× the cutoff. Contrived DBS samples were not utilized for the clinical correlation study.

## Results and Discussion

### Assessment of DBS analytical measurement range and imprecision

#### DBS limit of blank (LOB)

In order to assess the detection capability of the assay with DBS extracts, guidance from CLSI EP17-A2 was utilize^25^. Two separate reagent lots were used to make 96 blank measurements on 6 contrived blood samples over a 4-day period. Two results were not included in the data analysis for each lot as the z-score for each of these results with respect to the remaining results were greater than 4.7 for both reagent lots. Using the mean and standard deviation of the remaining blank results (n = 94) as well as a normal distribution multiplier, the LOB for DBS extracts was determined to be 0.111 U/mL (Supplementary Sect. 1).

#### DBS limit of detection (LOD)

The limit of detection (LOD) was assessed using 5 contrived blood samples in accordance with CLSI EP05-A3 and CLSI EP17-A2 guidance (Supplementary Sect. 2)^25,26^. The samples utilized had mean results (across two reagent lots) that were expected to be close to the clinical cutoff (0.146–0.531 U/mL). Pooled standard deviations were calculated for the two different reagent lots using CLSI EP17-A2 guidance^25^. The first reagent lot produced a pooled standard deviation of 0.0419 U/mL while the second demonstrated a standard deviation of 0.0346 U/mL. Using the larger standard deviation (0.0419 U/mL), a normal distribution multiplier, and the reported LOB (0.111 U/mL) the LOD for DBS extracts was determined to be 0.180 U/mL.

#### DBS limit of quantitation (LOQ)

Measurements to determine the limit of quantitation (LOQ) of DBS extracts utilized 14 contrived blood samples covering an appropriate range of concentrations (0.0528–0.648 U/mL) These samples were extracted and measured in triplicate over a five-day period (15 total measurements for each level) using two different reagent lots on a single instrument. For this study, the target CV and bias were set to 25.0% based on FDA guidance for ligand binding assays at the lower limit of quantitation^27^. Following collection of data, the imprecision profiles were analyzed using the Limit of Quantitation module in EP Evaluator (Supplementary Sect. 3). These results indicated an LOQ of 0.0873 U/mL for the first reagent lot while the second reagent lot demonstrated an LOQ of 0.0736 U/mL. In addition, acceptable biases less than ± 25.0% were observed for all levels greater than the DBS LOD. As both imprecision and bias results indicate an LOQ less than the observed LOD, the LOQ for DBS extracts is in practice equivalent to the LOD—0.180 U/mL^25^.

Investigation of the DBS assay’s LOB, LOD, and LOQ indicates that a lower reporting limit can be achieved for DBS extracts. This may be attributable to the reduction of sample dependent matrix effects as a result of ~ tenfold extraction (dilution) with the approved assay diluent^28^. As a result of the increased sensitivity, a clinical cutoff value below the EUA approved assay’s LOQ for serum and plasma can be implemented for DBS extracts, enabling assessment of categorical agreement between venous and DBS samples near the serum cutoff (0.8 U/mL).

#### DBS linearity

Linearity of the assay with DBS samples was assessed using 11 antibody concentrations made through serum sample admixtures in 10% increments followed by contrivance into DBS (Supplementary Sect. 4). Initial assessment of linearity indicated acceptable biases (± 20.0%) from 0.0667 to 147 U/mL with 147 U/mL being the highest concentration tested. This study was repeated with samples of higher concentration to extend the measurement range to 250 U/mL in order to match the reporting limit of un-diluted samples for the EUA approved assay. For this second study, samples with extracted concentrations that initially measured > 250 U/mL were manually diluted tenfold with Universal Diluent and re-measured as indicated in the assay’s package insert^23^. Antibody concentrations for this second study ranged from 0.0974 to 323 U/mL and targets were determined by using a linear fit of all data points.

#### DBS clinical cutoff

The clinical cutoff, used to assign DBS results as negative or positive for antibodies, for DBS samples was created using results from self-collected samples from donors confirmed to be seronegative (n = 77, Supplementary Sect. 5). The standard deviation of these results (0.0405 U/mL) was multiplied by 3 and added to the mean (0.0625 U/mL) to give a value of 0.184 U/mL. The DBS clinical cutoff for positivity was then set to ≥ 0.185 U/mL, where all results less than 0.185 are reported as negative.

#### DBS analytical measurement range

Evaluation of the detection capability of the assay with DBS extracts indicates that sample concentrations can be measured from 0.180 U/mL (LOD/LOQ) to 250 U/mL (upper reporting limit of assay) with a clinical negative/positive antibody cutoff concentration of 0.185 U/mL. This DBS range represents a calculated serum measurement range of 2.6 to 3570 U/mL. Although dilution of samples is performed for venous serum and plasma samples for the EUA approved in order to extend the reporting limit, dilution of DBS samples is not currently utilized due to the relatively wider concentration range that can be reported for DBS samples (as a result of pre-dilution through extraction).

#### Imprecision of DBS samples

Assay imprecision was assessed for DBS extracts using two reagent lots over a 4-day period with 6 contrived blood samples that covered a range of antibody concentrations (0.504–156 U/mL). A total of 16 replicates for each sample were measured (Supplementary Sect. 6). For both reagent lots, the CVs observed for repeatability and within-laboratory imprecision were less than 15.0% which meets FDA specifications for ligand binding assays (Table 2)^27^. All samples had a total categorical agreement of 100.0%.

**Table 2:** Imprecision of extracted DBS samples (reagent lots 53688601/54862501).

Sample	Mean (U/ml)	Repeatability (%)	Within-laboratory (%)	Total agreement (%)
1	0.530/0.504	7.6/6.1	10.9/9.4	100.0/100.0
2	0.682/0.661	8.5/7.6	11.3/10.1	100.0/100.0
3	5.97/5.93	7.7/7.6	14.7/12.8	100.0/100.0
4	15.3/15.3	6.9/6.1	8.6/8.0	100.0/100.0
5	61.8/61.9	12.5/13.2	12.5/13.2	100.0/100.0
6	156/156	7.7/7.9	8.7/7.9	100.0/100.0

### Clinical correlation study

Following collection and measurement of samples, 6 of the 84 presumed negative donors had venous serum, self-collected DBS samples, and professionally collected DBS samples measure positive for SARS-CoV-2 antibodies despite having negative RT-PCR results at the time of serological specimen collection (Supplementary Sect. 7). The serum results for these donors ranged from 8.81 to 1170 U/mL where the clinical cutoff for serum samples is 0.800 U/mL. These results suggest previous (asymptomatic) infection or unreported vaccination. To confirm these results, the serum samples for these donors were measured using three additional EUA approved serology assays (Roche Elecsys Anti-SARS-CoV-2, DiaSorin Liaison SARS-CoV-2 S1/S2 IgG and DiaSorin Liaison SARS-CoV-2 IgM)^29^. All six donors had serum results measure as positive on at least one additional assay and as such results were excluded from qualitative and quantitative analysis. All remaining results obtained were analyzed qualitatively using the established clinical cutoff for DBS extracts (0.185 U/mL) and quantitatively through correlative analysis.

**Figure 2:**
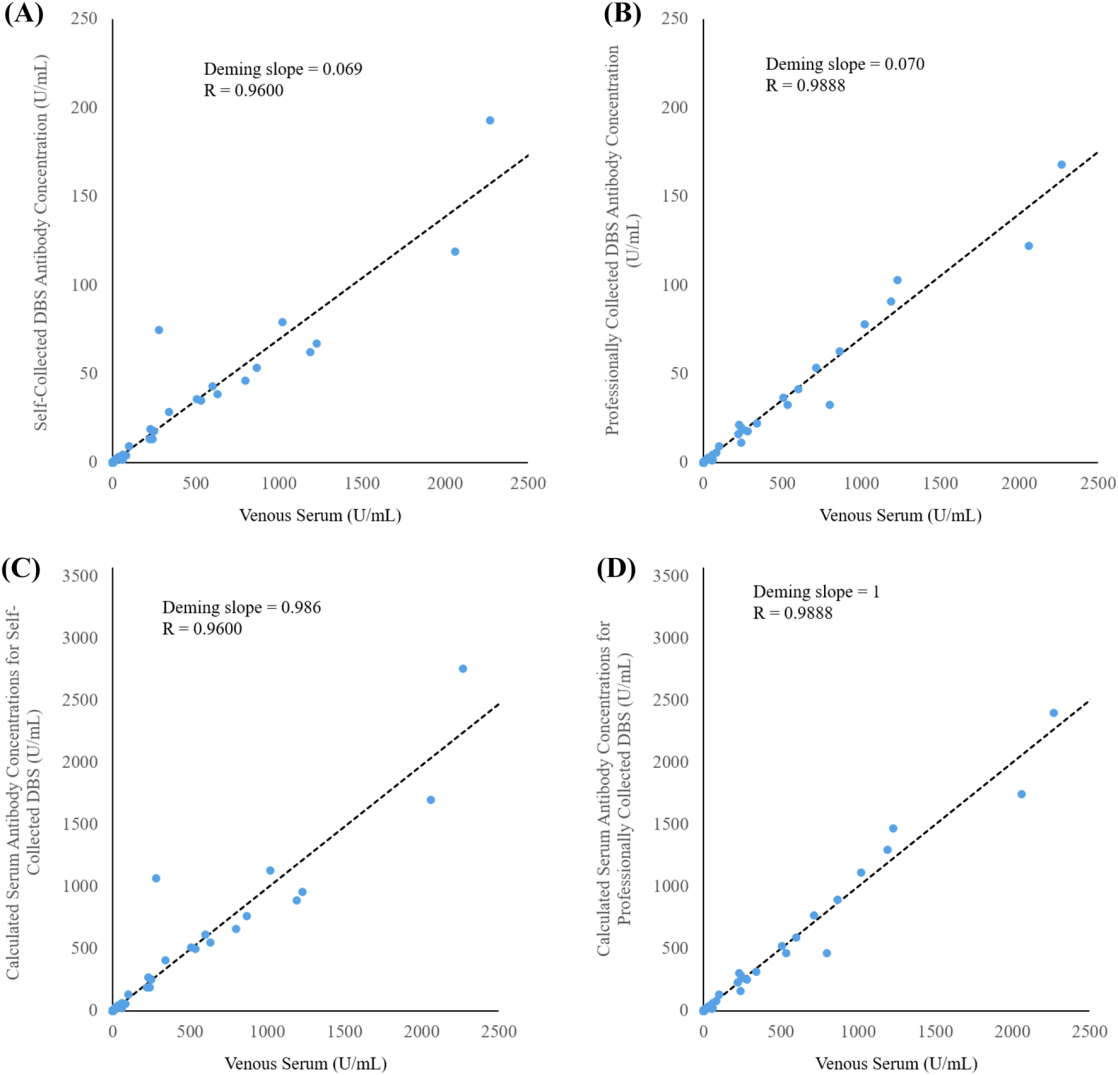
Serum antibody concentrations compared to (**A**) self-collected DBS sample antibody levels and (**B**) professionally collected DBS sample antibody levels. Calculated serum concentrations are also shown with respect to DBS concentrations (by dividing DBS results by 0.070) for both (**C**) self-collected and (**D**) professionally collected samples.

Comparison of serum samples with DBS obtained through self-collection demonstrated a high degree of agreement (R = 0.9600) and Deming slope of 0.069 (n = 108), which is attributed to dilution of the sample through the extraction process as well as precise but incomplete recovery of antibodies (Fig. 2A,C). Two donors with negative venous serum results had self-collected DBS samples that measured positive for antibodies (results within 3.5× the DBS clinical cutoff, Supplementary Sect. 5). As a result of these two false positives as well as a false negative donor with unmeasurable antibody results for both serum and DBS, qualitative total categorical agreement of self-collected DBS samples compared to venous serum results was 98.1% (Table 3A). In addition, the negative percent agreement (NPA) and positive percent agreement PPA were found to be 97.4% and 100.0%, respectively. When compared to RT-PCR results, qualitative categorical agreement was 97.2% with an NPA and PPA of 97.3% and 97.1%, respectively (Table 3B). Results below and above the DBS measurement range were interpreted as negative and positive, respectively.

**Table 3:** Qualitative comparisons of self-collected DBS results to (A) serum and (B) RT-PCR results and professionally collected DBS results to (C) serum and (D) RT-PCR results.

**A)**			**Serum results**			
			**−**	+		
	Self-collected DBS results	−	74	0	74	NPV = 100.0%
		+	2	32	34	PPV = 94.1%
			76	32	108	Total agreement:
			NPA = 97.4%	PPA = 100.0%		98.1%

**B)**			**RT-PCR results**			
			**−**	+		
	Self-collected DBS results	**−**	73	1	74	NPV = 98.6%
		+	2	33	35	PPV = 94.3%
		Total	75	34	109	Total agreement:
			NPA = 97.3%	PPA = 97.1%		97.2%

**C)**			**Serum results**			
			**−**	+		
	Professionally collected DBS results	**−**	79	0	79	NPV = 100.0%
		+	0	32	32	PPV = 100.0%
			79	32	111	Total agreement:
			NPA = 100.0%	PPA = 100.0%		100.0%

**D)**			**RT-PCR results**			
			**−**	+		
	Professionally collected DBS results	−	78	1	79	NPV = 98.7%
		+	0	32	32	PPV = 100.0%
			78	33	111	Total agreement:
			NPA = 100.0%	PPA = 97.0%		99.1%

When quantitatively comparing professionally-collected DBS samples to serum results (n = 106), results were similar to self-collected results with a correlation coefficient (R) of 0.9888 and a Deming slope of 0.070 (Fig. 2B,D). Total qualitative categorical agreement (n = 111) to venous serum results was 100.0% (Table 3C). When compared to RT-PCR results, qualitative categorical agreement was 99.1% with an NPA and PPA of 100.0% and 97.0%, respectively (Table 3D). These results met FDA guidance at the time of these studies (NPA ≥ 95.0%, PPA ≥ 90.0%)^22^.

### Robustness studies

In order to evaluate sample stability during the shipping process, simulated shipping studies were performed in accordance with ISTA 7D guidance as recommended by the FDA^22,30^. Contrived DBS samples were prepared in triplicate and split between three storage conditions: room temperature (20–25 °C), a simulated winter and summer shipping excursions (Supplementary Sect. 8). Following completion of the excursions, samples were measured in a single measurement run where results from samples stored in parallel at room temperature were used as baseline results.

Additional robustness and analytical interference studies were performed to stress different aspects of the DBS sample collection process as well as the influence of several endogenous or exogenous interferences (Supplementary Sects. 9 and 10). For acceptance of results, a mean bias of ± 20.0% was used as quantitative acceptance following the FDA guidance for ligand binding assays^27^. For qualitative assessment, a total categorical agreement of 95.0% was utilized based on guidance from the FDA’s Home Specimen Collection Serology Template^22^.

#### DBS shipping stability

For shipping excursion studies, contrived blood samples were created in triplicate and split into three shipping conditions: baseline ambient (20–25 °C), winter and summer excursions (Supplementary Sect. 8). Results less than 0.180 U/mL (DBS assay LOQ) were included in qualitative analysis but excluded from quantitative analysis. Overall, the sample results from winter and summer excursions both demonstrated total categorical agreement of 97.4% with mean biases of 6.0% and − 0.5%, respectively (Table 4). These results indicate that samples are stable from the time of collection in an individual’s home until received in the laboratory.

**Table 4:** Summary of DBS robustness study results.

Excursion	Hour(s)	Mean bias (%)	n^a^	Categorical agreement (%)	n
Winter	56	6.0	58	97.4	76
Summer	56	− 0.5	57	97.4	76
Alternate drying times	0	− 32.5	12	95.0	20
	1	1.3	12	95.0	20
	22	1.3	12	100.0	20
Humid drying	1	− 13.3	12	95.0	20
	3	− 22.2	12	95.0	20
	22	− 44.6	12	95.0	20
**Contamination**					
Alcohol exposure		− 11.0	13	100.0	20
Pressing finger to card		1.3	14	95.0	20

^a^Results below LOD were excluded from bias analysis.

#### Stress testing the collection process

For robustness studies, results that were found to be less than the assay’s DBS LOQ were excluded from bias analysis but included in qualitative analysis. Investigation of drying times compared to the recommended drying time of 3 h (prior to sample storage) was performed. Samples that were immediately stored after contrived blood was added to the card had a categorical agreement of 95.0% and mean bias of − 32.5%. Results from samples that were dried for 1 and 22 h demonstrated categorical agreements of 95.0 and 100.0%, respectively with mean bias of 1.3% for both (Table 4). Effects of drying samples in a humid environment (40 °C, > 95% relative humidity) were also investigated^22^. All results observed had a categorical agreement of 95.0%, but the magnitude of the bias increased from − 13.3% for 1 h to − 44.6% for 22 h of humid drying.

Contamination as a result of potential errors in the collection process was investigated (Table 4, Supplementary Sect. 9). Exposure of DBS spots to alcohol, which may occur following finger sterilization without allowing the fingertip to dry, demonstrated a categorical agreement of 100.0% and mean bias of − 11.0%. Contamination of the DBS card by an unsterilized finger prior to collection demonstrated a categorical agreement of 95.0% and mean bias of 1.3%. Although all robustness studies had a total categorical agreement greater than or equal to 95.0%, biases less than ± 20.0% were observed in some instances. As these studies are not exhaustive of all possible contaminants, proper instruction materials must be provided to the patient in order to insure the collection of a sample of sufficient quality for measurement.

#### Analytical interference studies

Several studies were performed to assess the effects of different endogenous and exogenous interferents on the measurement of SARS-CoV-2 antibodies from DBS samples (Supplementary Sect. 10). Results from all interference studies had a categorical agreement greater than or equal to 95.0% to baseline measurements (Table 5). The exogenous interferents tested had mean biases less than ± 5.0% while most endogenous interferents tested had a mean bias result less than ± 10.0% with the exception of excess protein (17.7%).

**Table 5:** Summary of DBS analytical interference results.

Analytical interferents	Mean bias (%)	n^a^	Categorical agreement (%)	n
**Endogenous interferents**				
Hemolysis (100%)	3.6	15	95.0	20
Triglycerides (3000 mg/dL)	4.5	18	96.7	30
Total protein (12 g/dL)	17.7	19	100.0	30
Conjugated bilirubin (20 mg/dL)	− 2.8	20	96.7	30
Unconjugated bilirubin (20 mg/dL)	− 8.7	20	100.0	30
**Exogenous interferents**				
Cerilliant mix 1	− 0.7	20	96.7	30
Cerilliant mix 2	2.5	20	100.0	30
Biotin (3510 ng/mL)	4.5	21	96.7	29

^a^Results below LOD were excluded from bias analysis.

### Immunization study

**Figure 3:**
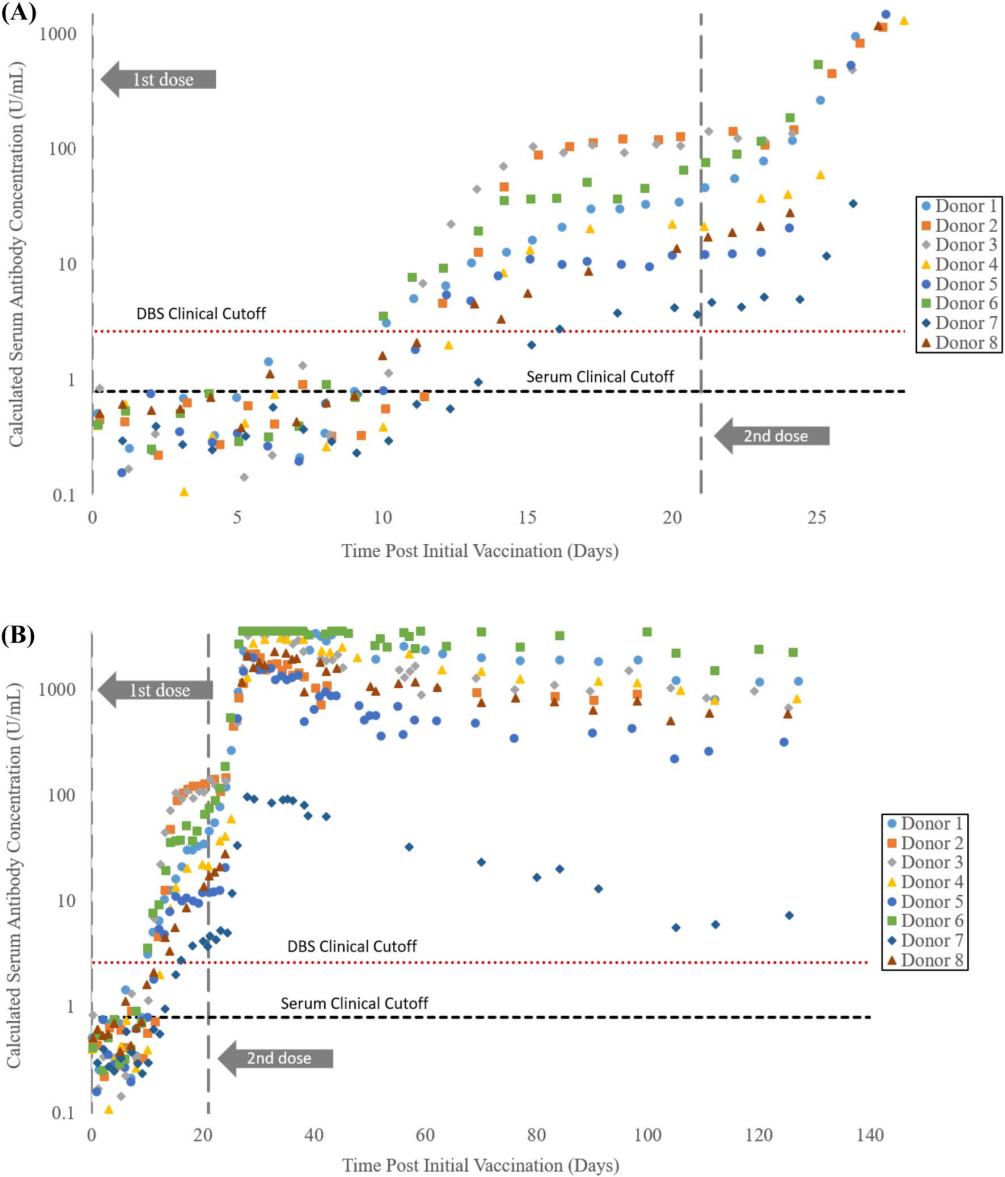
SARS-CoV-2 antibody levels measured from DBS samples following (**A**) pre-dose (Day 0) through receipt of second vaccine dose (Day 21) to day 28 and (**B**) through Day 132. Y-axes are displayed logarithmically with the left axis representing calculated serum levels (by dividing DBS results by 0.070).

Application of self-collected DBS was performed with serial monitoring of SARS-CoV-2 antibody levels following immunization in 8 donors. Donors periodically performed self-collection of DBS samples pre-vaccination through 19 weeks following initial vaccination. All donors reported receiving the Pfizer-BioNTech COVID-19 vaccine with the second dose occurring exactly 3 weeks following the first dose. As can be seen in Fig. 3A below, all donors had negative DBS results for samples collected within the first 9 days following initial immunization. Antibody levels for each donor rose above the DBS cutoff of 0.185 U/mL between days 10 and 16. Antibody levels increased rapidly following the second vaccination dose (Fig. 3B), with several donors reaching levels greater than the DBS reporting limit (250 U/mL which is calculated to be 3570 U/mL in serum)^31,32^. Dramatically lower antibody levels were observed for Donor 7, likely a result of the immunosuppressive medication the donor reported taking for a chronic condition^33^.

## Conclusions

The thorough results provided herein demonstrate the robustness of measuring SARS-CoV-2 antibodies using DBS samples. Although DBS samples are diluted through the extraction process (and as a result of incomplete antibody recovery), this approach also has advantages. Using Roche’s Universal Diluent as the extraction buffer, sample-to-sample matrix effects were reduced and a lower (absolute) reporting limit was achieved (LOQ of 0.180 U/mL for DBS samples). In addition, the dilution of the sample allowed a higher relative measurement range to be demonstrated as a DBS samples with a concentration of 250 U/mL DBS sample (upper limit of quantitation for un-diluted venous samples) would be greater than 3500 U/mL in serum. These results, as well as the high correlation to venous serum results, allowed the assay to be used to demonstrate antibody monitoring over time through at-home DBS self-collections. Ultimately, DBS samples could serve as an important tool for regular antibody monitoring and scheduling of immunization boosters when antibody levels inferring protective immunity is more fully understood in the future.

## Supplementary Information

Supplementary Information.

## References

[1] Qi L (2020). Factors associated with the duration of viral shedding in adults with COVID-19 outside of Wuhan, China: A retrospective cohort study. Int. J. Infect. Dis..

[2] World Health Organization (WHO). Infection prevention and control of epidemic- and pandemic-prone acute respiratory infections in health care. *WHO Guidel.* 1–156. https://www.who.int/publications/i/item/infection-prevention-and-control-of-epidemic-and-pandemic-prone-acute-respiratory-infections-in-health-care (2014). 24983124

[3] Ghaffari A, Meurant R, Ardakani A (2020). COVID-19 serological tests: How well do they actually perform?. Diagnostics.

[4] Wang YC (2020). Current diagnostic tools for coronaviruses—From laboratory diagnosis to POC diagnosis for COVID-19. Bioeng. Transl. Med..

[5] Alessandro, S. & Crotty, S. Adaptive immunity to SARS-CoV-2 and COVID-19. *Ann. Oncol*. 19–21 (2020).

[6] Khoury DS (2021). Neutralizing antibody levels are highly predictive of immune protection from symptomatic SARS-CoV-2 infection. Nat. Med..

[7] U.S. Food & Drug Administration. Antibody (Serology) Testing for COVID-19_ Information for Patients and Consumers_FDA.

[8] Zakaria R, Allen KJ, Koplin JJ, Roche P, Greaves RF (2016). Advantages and challenges of dried blood spot analysis by mass spectrometry across the total testing process. Ejifcc.

[9] Amini F (2021). Reliability of dried blood spot (DBS) cards in antibody measurement: A systematic review. PLoS ONE.

[10] Zava TT, Zava DT (2020). Validation of dried blood spot sample modifications to two commercially available COVID-19 IgG antibody immunoassays. Bioanalysis.

[11] Selva L (2013). Detection of *Streptococcus pneumoniae* and *Haemophilus influenzae* type B by real-time PCR from dried blood spot samples among children with pneumonia: A useful approach for developing countries. PLoS ONE.

[12] Chang M, Johnston S, Seilie AM, Hergott D, Murphy SC (2021). Application of dried blood spot sample pooling strategies for Plasmodium 18S rRNA biomarker testing to facilitate identification of infected persons in large-scale epidemiological studies. Malar. J..

[13] Tuaillon E (2020). Dried Blood Spot Tests for the Diagnosis and Therapeutic Monitoring of HIV and Viral Hepatitis B and C. Front. Microbiol..

[14] Parker SP, Cubitt WD (1999). The use of the dried blood spot sample in epidemiological studies. J. Clin. Pathol..

[15] Mulchandani R (2021). Use of dried blood spot samples for SARS-CoV-2 antibody detection using the Roche Elecsys® high throughput immunoassay. J. Clin. Virol..

[16] Brinc D (2021). Evaluation of dried blood spot testing for SARS-CoV-2 serology using a quantitative commercial assay. Viruses.

[17] Turgeon CT (2021). Detection of SARS-CoV-2 IgG antibodies in dried blood spots. Diagn. Microbiol. Infect. Dis..

[18] Turgeon CT (2021). Validation of a multiplex flow immunoassay for detection of IgG antibodies against SARS-CoV-2 in dried blood spots. PLoS ONE.

[19] Weisser H (2021). Evaluation of dried blood spots as alternative sampling material for serological detection of anti-SARS-CoV-2 antibodies using established ELISAs. Clin. Chem. Lab. Med..

[20] Beyerl J (2021). A dried blood spot protocol for high throughput analysis of SARS-CoV-2 serology based on the Roche Elecsys anti-N assay. EBioMedicine.

[21] Cholette F (2021). Dried blood spot specimens for SARS-CoV-2 antibody testing: A multi-site, multi-assay comparison. PLoS ONE.

[22] U.S. Food & Drug Administration. Home Specimen Collection Serology Template for Fingerstick Dried Blood Spot. https://www.fda.gov/medical-devices/coronavirus-disease-2019-covid-19-emergency-use-authorizations-medical-devices/vitro-diagnostics-euas (2020) (Accessed September 30, 2021).

[23] Roche Diagnostics. *Elecsys Anti-SARS-CoV-2 S Elecsys Anti-SARS-CoV-2 S*. (2020).

[24] U.S. Food & Drug Administration. In Vitro Diagnostics EUAs—Molecular Diagnostic Tests for SARS-CoV-2|FDA. https://www.fda.gov/medical-devices/coronavirus-disease-2019-covid-19-emergency-use-authorizations-medical-devices/in-vitro-diagnostics-euas-molecular-diagnostic-tests-sars-cov-2 (2021) (Accessed March 18, 2022).

[25] Clinical and Laboratory Standards Institute. *EP17-A2 Evaluation of Detection Capability for Clinical Laboratory Measurement Procedures; Approved Guideline*. (2012).

[26] Clinical and Laboratory Standards Institute. *EP05-A3 Evaluation of precision of quantitative measurement procedures*. *Clinical and Laboratory Standards Institute* vol. 34 (2014).

[27] Food and Drug Administration. Bioanalytical method validation guidance for industry. *Food Drug Adm.* (2018).

[28] Wood WG (1991). ‘Matrix effects’ in immunoassays. Scand. J. Clin. Lab. Invest. Suppl..

[29] US Food & Drug Administration (2020). EUA authorized serology test performance. FDA..

[30] International Safe Transit Association. *Temperature Test for Transport Packaging; ISTA 7 Series Developemt Test Procedure*. (2013).

[31] Livingston EH (2021). Necessity of 2 doses of the Pfizer and Moderna COVID-19 vaccines. JAMA.

[32] Dagan N (2021). BNT162b2 mRNA COVID-19 vaccine in a nationwide mass vaccination setting. N. Engl. J. Med..

[33] Negahdaripour M (2021). Administration of COVID-19 vaccines in immunocompromised patients. Int. Immunopharmacol..

