# Supplementary material for: Monitoring of SARS-CoV-2 antibodies using dried blood spot for at-home collection
Source: Sci Rep. 2022 Apr 6;12:5812. doi: 10.1038/s41598-022-09699-4 (PMC8985748; doi:10.1038/s41598-022-09699-4)
Supplement: Supplementary file 1 — Supplementary Information. [file 41598_2022_9699_MOESM1_ESM.docx]

**Supplementary Information**

Monitoring of SARS-CoV-2 Antibodies Using Dried Blood Spot for At-Home Collection

Peyton K. Miesse^1^, Bradley B. Collier^1,*^, Russell P. Grant^1^

^1^Center for Esoteric Testing, Laboratory Corporation of America Holdings, Burlington, North Carolina, USA

^*^collib7@labcorp.com

# Limit of Blank (LOB)

Six pre-pandemic serum samples were utilized to assess the assay’s limit of blank (LOB) following creation into contrived blood samples for spotting and extraction from DBS cards. This produced 16 total measurements for each sample for a total of 96 “blank” measurements for each lot. A single instrument was used for this study. In accordance with CLSI EP17-A2 guidance, the mean ($M_{blank}$) and standard deviation (${SD}_{blank}$) of all samples was determined across two reagent lots (1). All results from sample 4 on Day 2, Run 1 were excluded as the z-score of these results was greater than 4.7 with respect to other results measured using the same lot. In addition to the mean and standard deviation, a multiplier ($c_{p}$) which represents the 95^th^ percentile of normal distribution was utilized. This multiplier was corrected for the use of the biased observed standard deviation estimate using equation 1 below where B represents the total number of blank results in the dataset (B = 94) and K represents the number of blank samples (K = 6).

$c_{p}=\frac{1.645}{1-(\frac{1}{4\left( B-K \right)})}$ (Equation 1)

$LOB= M_{blank}+c_{p} {SD}_{blank}$ (Equation 2)

The mean and standard deviation observed for reagent lot 53688601 were 0.0617 and 0.0296 U/mL, respectively (Table 1). With a multiplier value of 1.6497, the calculated LOB for reagent lot 53688601 was 0.111 U/mL. For reagent lot 54862501, the mean and standard deviation was determined to be 0.0417 and 0.0298 U/mL, respectively, giving a LOB of 0.0908 U/mL (Table 2). As the calculated LOB for reagent lot 53688601 is greater, it represents the LOB listed in the manuscript.

Table 2: Pre-pandemic DBS limit of blank samples measured with reagent lot 53688601

|  | Rep. | Pre-Pandemic Sample Results (U/mL) | | | | | |
| --- | --- | --- | --- | --- | --- | --- | --- |
|  |  | 1 | 2 | 3 | 4 | 5 | 6 |
| Day 1, Run 1 | 1 | 0.0555 | 0.066 | 0.0557 | 0.0711 | 0.162 | 0.0862 |
|  | 2 | 0.118 | 0.143 | 0.149 | 0.0723 | 0.176 | 0.0101 |
| Day 1, Run 2 | 3 | 0.0738 | 0.0560 | 0.0624 | 0.0565 | 0.0813 | 0.0753 |
|  | 4 | 0.0748 | 0.0632 | 0.0449 | 0.0512 | 0.0515 | 0.0696 |
| Day 2, Run 1 | 5 | 0.0446 | 0.0385 | 0.0530 | 0.235* | 0.0702 | 0.0488 |
|  | 6 | 0.0974 | 0.0852 | 0.0994 | 0.209* | 0.0825 | 0.0696 |
| Day 2, Run 2 | 7 | 0.0496 | 0.0488 | 0.0462 | 0.0468 | 0.0475 | 0.0403 |
|  | 8 | 0.0666 | 0.0615 | 0.0586 | 0.0583 | 0.0705 | 0.0683 |
| Day 3, Run 1 | 9 | 0.0386 | 0.0231 | 0.0598 | 0.0483 | 0.0575 | 0.0540 |
|  | 10 | 0.0566 | 0.0223 | 0.0273 | 0.0414 | 0.0213 | 0.0369 |
| Day 3, Run 2 | 11 | 0.0479 | 0.0609 | 0.0590 | 0.0395 | 0.0425 | 0.0502 |
|  | 12 | 0.0674 | 0.0670 | 0.0615 | 0.0540 | 0.0652 | 0.0562 |
| Day 4, Run 1 | 13 | 0.0559 | 0.0501 | 0.0622 | 0.0616 | 0.0610 | 0.0577 |
|  | 14 | 0.0255 | 0.176 | 0.0269 | 0.0213 | 0.0420 | 0.0430 |
| Day 4, Run 2 | 15 | 0.0436 | 0.0409 | 0.0574 | 0.0373 | 0.0445 | 0.0602 |
|  | 16 | 0.0699 | 0.0562 | 0.0722 | 0.0584 | 0.0695 | 0.0724 |
| *Results were excluded from analysis as z-scores with respect to remaining data were greater than 4.97. | | | | | | | |

Table 3: Pre-pandemic DBS limit of blank samples measured with reagent lot 54862501

|  | Rep. | Pre-Pandemic Sample Results (U/mL) | | | | | |
| --- | --- | --- | --- | --- | --- | --- | --- |
|  |  | 1 | 2 | 3 | 4 | 5 | 6 |
| Day 1, Run 1 | 1 | 0.0253 | 0.0408 | 0.0290 | 0.0409 | 0.141 | 0.0590 |
|  | 2 | 0.116 | 0.138 | 0.145 | 0.0728 | 0.179 | 0.0801 |
| Day 1, Run 2 | 3 | 0.0275 | 0.0212 | 0.0278 | 0.0288 | 0.0399 | 0.0380 |
|  | 4 | 0.0600 | 0.0463 | 0.0340 | 0.0376 | 0.0368 | 0.0442 |
| Day 2, Run 1 | 5 | 0.0255 | 0.0378 | 0.0561 | 0.227* | 0.0695 | 0.0467 |
|  | 6 | 0.0693 | 0.0602 | 0.0782 | 0.184* | 0.0566 | 0.0536 |
| Day 2, Run 2 | 7 | 0.0246 | 0.0197 | 0.0248 | 0.0304 | 0.0315 | 0.0383 |
|  | 8 | 0.0275 | 0.0285 | 0.0323 | 0.0336 | 0.0336 | 0.0227 |
| Day 3, Run 1 | 9 | 0.0318 | 0.0280 | 0.0281 | 0.0328 | 0.0209 | 0.0292 |
|  | 10 | 0.0249 | 0.0321 | 0.0289 | 0.0316 | 0.037 | 0.0217 |
| Day 3, Run 2 | 11 | 0.0319 | 0.0455 | 0.057 | 0.0291 | 0.0248 | 0.0232 |
|  | 12 | 0.0262 | 0.0238 | 0.0331 | 0.0280 | 0.0230 | 0.0175 |
| Day 4, Run 1 | 13 | 0.0361 | 0.0356 | 0.0311 | 0.0383 | 0.0356 | 0.0247 |
|  | 14 | 0.0287 | 0.125 | 0.0367 | 0.0261 | 0.0374 | 0.0290 |
| Day 4, Run 2 | 15 | 0.0234 | 0.0165 | 0.0120 | 0.0217 | 0.0275 | 0.0380 |
|  | 16 | 0.0288 | 0.0227 | 0.0227 | 0.0181 | 0.0355 | 0.0508 |
| * Results were excluded from analysis as z-scores with respect to remaining data were greater than 4.78. | | | | | | | |

# DBS Limit of Detection (LOD)

Five (5) contrived DBS samples were created to have concentrations that were expected to cover the clinical cutoff range (0.126 to 0.415 U/mL), which allows assessment of the assay’s LOB. DBS extracted and measured in duplicate for each run with 2 runs being performed per day over 4 days giving 16 total measurements for each sample. A single instrument was used for this study. In accordance with CLSI EP05-A3 and EP17-A2 guidance, the within laboratory standard deviation of all samples was determined across two reagent lots (1). The pooled standard deviation (${SD}_{L}$) was then determined using:

${SD}_{L}=\sqrt{\frac{\Sigma_{ⅈ=1}^{J}\left( n_{i}-1 \right)SD_{i}^{2}}{\Sigma_{ⅈ=1}^{J}\left( n_{i}-1 \right)}}$ (Equation 3)

where $SD_{i}$ represents the SD of all results for the *i*th low level sample, $n_{i}$ is the number of results for the *i*th low level sample ($n_{i}=16$), and $J$ is the number of low level samples ($J=5$). Using equation 3, the pooled standard deviation for reagent lot 53688601 was found to be 0.0419 U/mL (Table 3) and 0.0346 U/mL for reagent lot 54862501 (Table 4). The larger of these values, 0.0419 U/mL was used with LOB results and a multiplier to determine the assay’s LOD:

$c_{p}= \frac{1.645}{1-\left( \frac{1}{4(L-J)} \right)}$ (Equation 4)

$LOD=LOB+c_{p}\mathrm{SD}_{L}$ (Equation 5)

where $L$ is the total number of all low level sample ($L=80$). The LOD was calculated with a multiplier of 1.6505, and the reported LOB (0.111 U/mL) to give a LOD of 0.180 U/mL.

Table 4: DBS Limit of detection results (reagent lot 53688601)

| Day # | Rep. | Low Concentration DBS Results (U/mL) | | | | |
| --- | --- | --- | --- | --- | --- | --- |
|  |  | 1 | 2 | 3 | 4 | 5 |
| Day 1, Run 1 | 1 | 0.170 | 0.182 | 0.279 | 0.325 | 0.382 |
|  | 2 | 0.158 | 0.171 | 0.294 | 0.341 | 0.329 |
| Day 1, Run 2 | 3 | 0.173 | 0.256 | 0.324 | 0.415 | 0.411 |
|  | 4 | 0.146 | 0.188 | 0.332 | 0.349 | 0.531 |
| Day 2, Run 1 | 5 | 0.244 | 0.149 | 0.352 | 0.322 | 0.451 |
|  | 6 | 0.248 | 0.215 | 0.275 | 0.408 | 0.391 |
| Day 2, Run 2 | 7 | 0.204 | 0.202 | 0.303 | 0.365 | 0.390 |
|  | 8 | 0.184 | 0.159 | 0.325 | 0.325 | 0.400 |
| Day 3, Run 1 | 9 | 0.154 | 0.197 | 0.297 | 0.346 | 0.373 |
|  | 10 | 0.169 | 0.153 | 0.300 | 0.338 | 0.370 |
| Day 3, Run 2 | 11 | 0.164 | 0.195 | 0.264 | 0.322 | 0.421 |
|  | 12 | 0.175 | 0.206 | 0.321 | 0.311 | 0.409 |
| Day 4, Run 1 | 13 | 0.170 | 0.181 | 0.265 | 0.320 | 0.354 |
|  | 14 | 0.149 | 0.22 | 0.264 | 0.349 | 0.311 |
| Day 4, Run 2 | 15 | 0.154 | 0.201 | 0.270 | 0.365 | 0.322 |
|  | 16 | 0.160 | 0.168 | 0.232 | 0.324 | 0.333 |
| Total Mean | | 0.176 | 0.190 | 0.294 | 0.345 | 0.386 |
| Total SD | | 0.0307 | 0.0277 | 0.0318 | 0.0304 | 0.0548 |
| Total CV | | 17.4% | 14.5% | 10.8% | 8.8% | 14.2% |
| Repeatability (Error) | SS | 0.00104 | 0.00739 | 0.00507 | 0.00737 | 0.0115 |
|  | DF | 8 | 8 | 8 | 8 | 8 |
|  | MS | 0.000130 | 0.000924 | 0.000634 | 0.000921 | 0.00143 |
|  | SD | 0.0114 | 0.0304 | 0.0252 | 0.0303 | 0.0379 |
| Run (Day) | SS | 0.00279 | 0.00298 | 0.00194 | 0.00355 | 0.0159 |
|  | DF | 4 | 4 | 4 | 4 | 4 |
|  | MS | 0.000699 | 0.000745 | 0.000485 | 0.000888 | 0.00398 |
|  | SD | 0.0264 | 0.0273 | 0.0220 | 0.0298 | 0.0631 |
| Day | SS | 0.0103 | 0.000693 | 0.00752 | 0.00214 | 0.0177 |
|  | DF | 2 | 2 | 2 | 2 | 2 |
|  | MS | 0.00513 | 0.000347 | 0.00376 | 0.00107 | 0.00883 |
|  | SD | 0.0716 | 0.0186 | 0.0613 | 0.0327 | 0.0940 |
| Variance | Error | 0.000130 | 0.000924 | 0.000634 | 0.000921 | 0.00143 |
|  | Run | 0.000284 | 0 | 0 | 0 | 0.00127 |
|  | Day | 1.11E-03 | 0 | 8.19E-04 | 4.51E-05 | 1.21E-03 |
| Within-Laboratory Precision | SD | 0.0390 | 0.0304 | 0.0381 | 0.0311 | 0.0626 |

Table 5: DBS Limit of detection results (reagent tot 54862501)

| Day # | Rep. | Low Concentration DBS Results (U/mL) | | | | |
| --- | --- | --- | --- | --- | --- | --- |
|  |  | 1 | 2 | 3 | 4 | 5 |
| Day 1, Run 1 | 1 | 0.160 | 0.174 | 0.250 | 0.296 | 0.343 |
|  | 2 | 0.169 | 0.183 | 0.294 | 0.342 | 0.321 |
| Day 1, Run 2 | 3 | 0.135 | 0.223 | 0.301 | 0.366 | 0.393 |
|  | 4 | 0.145 | 0.199 | 0.292 | 0.317 | 0.487 |
| Day 2, Run 1 | 5 | 0.234 | 0.181 | 0.308 | 0.319 | 0.419 |
|  | 6 | 0.261 | 0.217 | 0.275 | 0.389 | 0.385 |
| Day 2, Run 2 | 7 | 0.169 | 0.192 | 0.277 | 0.312 | 0.386 |
|  | 8 | 0.168 | 0.176 | 0.294 | 0.311 | 0.375 |
| Day 3, Run 1 | 9 | 0.126 | 0.202 | 0.275 | 0.313 | 0.350 |
|  | 10 | 0.173 | 0.195 | 0.306 | 0.337 | 0.371 |
| Day 3, Run 2 | 11 | 0.155 | 0.166 | 0.239 | 0.290 | 0.415 |
|  | 12 | 0.142 | 0.213 | 0.284 | 0.299 | 0.389 |
| Day 4, Run 1 | 13 | 0.159 | 0.214 | 0.240 | 0.326 | 0.329 |
|  | 14 | 0.126 | 0.225 | 0.254 | 0.326 | 0.320 |
| Day 4, Run 2 | 15 | 0.142 | 0.196 | 0.235 | 0.322 | 0.302 |
|  | 16 | 0.123 | 0.196 | 0.228 | 0.305 | 0.319 |
| Total Mean | | 0.162 | 0.197 | 0.272 | 0.323 | 0.369 |
| Total SD | | 0.0376 | 0.0180 | 0.0272 | 0.0257 | 0.0479 |
| Total CV | | 23.3% | 9.1% | 10.0% | 8.0% | 13.0% |
| Repeatability (Error) | SS | 0.00224 | 0.00257 | 0.00341 | 0.00512 | 0.0060 |
|  | DF | 8 | 8 | 8 | 8 | 8 |
|  | MS | 0.000280 | 0.000321 | 0.000426 | 0.000639 | 0.00075 |
|  | SD | 0.0167 | 0.0179 | 0.0206 | 0.0253 | 0.0274 |
| Run (Day) | SS | 0.00694 | 0.00191 | 0.00172 | 0.00340 | 0.0140 |
|  | DF | 4 | 4 | 4 | 4 | 4 |
|  | MS | 0.00174 | 0.000479 | 0.000429 | 0.000850 | 0.00351 |
|  | SD | 0.0417 | 0.0219 | 0.0207 | 0.0292 | 0.0593 |
| Day | SS | 0.00795 | 0.000430 | 0.00403 | 0.000890 | 0.00956 |
|  | DF | 2 | 2 | 2 | 2 | 2 |
|  | MS | 0.00397 | 0.000213 | 0.00201 | 0.000445 | 0.00478 |
|  | SD | 0.0630 | 0.0146 | 0.0449 | 0.0211 | 0.0692 |
| Variance | Error | 0.000280 | 0.000321 | 0.000426 | 0.000639 | 0.000749 |
|  | Run | 7.28E-04 | 7.87E-05 | 1.65E-06 | 1.05E-04 | 1.38E-03 |
|  | Day | 0.000559 | 0 | 0.000396 | 0.00 | 0.000318 |
| Within-Laboratory Precision | SD | 0.0396 | 0.0200 | 0.0287 | 0.0273 | 0.0495 |

# **DBS Limit of Quantitation (LOQ)**

Limit of quantitation (LOQ) studies utilized 14 levels of contrived blood samples that ranged from less than the LOB and ranges that were expected to cover the clinical cutoff (0.0528 to 0.648 U/mL). DBS samples were extracted and measured in triplicate over a five-day period (total of 15 measurements per level). A single instrument was used for this study. Data was analyzed using the Limit of Quantitation module in EP Evaluator where the imprecision profile is fit to the equation CV = A + B x (1 / mean of measured values). The LOQ is the point where the upper 95% confidence interval of this fit is equal to the target CV. For this application, the target CV was set to 25% based on the FDA guidance for ligand binding assays at the lower limit of quantitation (2). Based on imprecision results, reagent lot 51595100 had a LOQ of 0.0873 U/mL while reagent lot 53688600 had a LOQ value of 0.0736 U/mL (Figures 2 and 3). Both of these results are less than the assay’s LOD.

In addition, LOQ results were evaluated in terms of bias where acceptable biases were also set to 25% based on FDA guidance. Bias results (Tables 5 and 6) indicated acceptable bias for all levels greater than the assay’s LOD. As both imprecision and bias results indicated an LOQ less than the assay’s LOD, the reported LOQ for the assay is in practice equivalent to the LOD (0.180 U/mL).

Figure 2: DBS Limit of quantitation imprecision results (reagent lot 51595100)


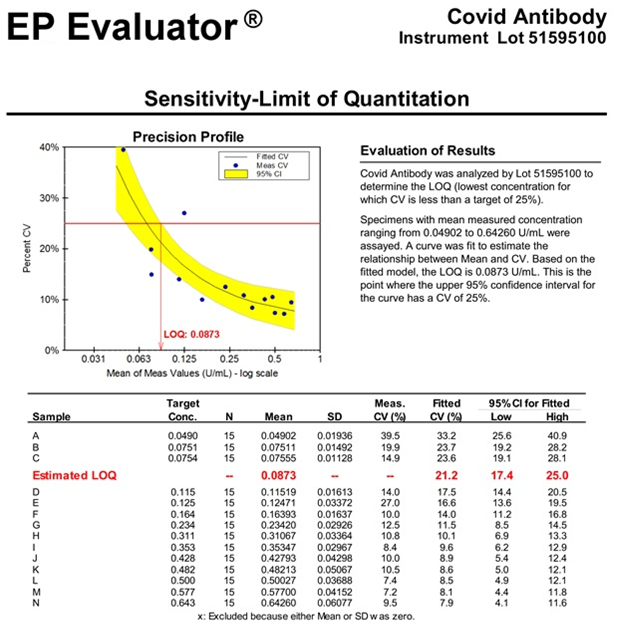


Table 6: DBS Limit of quantitation bias results (reagent lot 51595100)

| Level | | LOQ Sample Results (U/mL) | | | | | | | | | | | | | | | | |  |
| --- | --- | --- | --- | --- | --- | --- | --- | --- | --- | --- | --- | --- | --- | --- | --- | --- | --- | --- | --- |
|  |  | Day 1 | | | Day 2 | | | Day 3 | | | Day 4 | | | Day 5 | | | Linearity | |  |
|  |  | 1 | 2 | 3 | 4 | 5 | 6 | 7 | 8 | 9 | 10 | 11 | 12 | 13 | 14 | 15 | Target | Bias |  |
| A | 0.0% | 0.0579 | 0.0274 | 0.0498 | 0.0575 | 0.032 | 0.055 | 0.0767 | 0.0725 | 0.0876 | 0.0404 | 0.0440 | 0.0336 | 0.0287 | 0.0505 | 0.0217 | 0.0682 | NA | |
| B | 2.5% | 0.0580 | 0.0681 | 0.0951 | 0.0669 | 0.0982 | 0.0649 | 0.0666 | 0.0859 | 0.0976 | 0.0926 | 0.0707 | 0.0796 | 0.0608 | 0.0581 | 0.0635 | 0.0823 | NA | |
| C | 5.0% | 0.0729 | 0.0813 | 0.0584 | 0.087 | 0.0904 | 0.0831 | 0.0874 | 0.0873 | 0.0714 | 0.0668 | 0.0869 | 0.0647 | 0.0656 | 0.0575 | 0.0705 | 0.0964 | NA | |
| D | 7.5% | 0.107 | 0.0908 | 0.131 | 0.127 | 0.140 | 0.0841 | 0.131 | 0.117 | 0.134 | 0.110 | 0.112 | 0.101 | 0.104 | 0.119 | 0.120 | 0.111 | NA | |
| E | 10% | 0.112 | 0.113 | 0.113 | 0.126 | 0.0987 | 0.118 | 0.139 | 0.128 | 0.110 | 0.104 | 0.120 | 0.125 | 0.241 | 0.111 | 0.112 | 0.125 | NA | |
| F | 20% | 0.167 | 0.161 | 0.154 | 0.161 | 0.169 | 0.194 | 0.185 | 0.171 | 0.180 | 0.150 | 0.163 | 0.125 | 0.160 | 0.169 | 0.150 | 0.181 | -9.6% | |
| G | 30% | 0.205 | 0.263 | 0.194 | 0.234 | 0.267 | 0.244 | 0.280 | 0.271 | 0.247 | 0.238 | 0.182 | 0.230 | 0.232 | 0.224 | 0.202 | 0.238 | -1.5% | |
| H | 40% | 0.289 | 0.470 | 0.303 | 0.369 | 0.299 | 0.316 | 0.319 | 0.313 | 0.400 | 0.291 | 0.293 | 0.310 | 0.316 | 0.275 | 0.294 | 0.294 | 10.0% | |
| I | 50% | 0.360 | 0.340 | 0.382 | 0.333 | 0.385 | 0.388 | 0.333 | 0.401 | 0.332 | 0.341 | 0.382 | 0.336 | 0.371 | 0.308 | 0.310 | 0.351 | 0.7% | |
| J | 60% | 0.447 | 0.468 | 0.625 | 0.472 | 0.452 | 0.427 | 0.489 | 0.425 | 0.432 | 0.320 | 0.400 | 0.421 | 0.438 | 0.430 | 0.358 | 0.407 | 8.1% | |
| K | 70% | 0.481 | 0.515 | 0.526 | 0.505 | 0.568 | 0.530 | 0.479 | 0.513 | 0.488 | 0.514 | 0.405 | 0.463 | 0.438 | 0.396 | 0.411 | 0.464 | 3.9% | |
| L | 80% | 0.443 | 0.514 | 0.587 | 0.497 | 0.492 | 0.509 | 0.463 | 0.539 | 0.533 | 0.477 | 0.467 | 0.520 | 0.477 | 0.468 | 0.518 | 0.520 | -3.9% | |
| M | 90% | 0.541 | 0.559 | 0.579 | 0.601 | 0.607 | 0.637 | 0.599 | 0.554 | 0.530 | 0.617 | 0.58 | 0.654 | 0.527 | 0.553 | 0.517 | 0.577 | 0.0% | |
| N | 100% | 0.735 | 0.666 | 0.622 | 0.643 | 0.701 | 0.634 | 0.628 | 0.636 | 0.700 | 0.616 | 0.718 | 0.653 | 0.547 | 0.637 | 0.503 | 0.634 | 1.4% | |
|  |  | Levels E and M used to set Target Values | | | | | | | | | | | | | | |  |  |  |

Figure 3: DBS Limit of quantitation imprecision results (reagent lot 53688600)


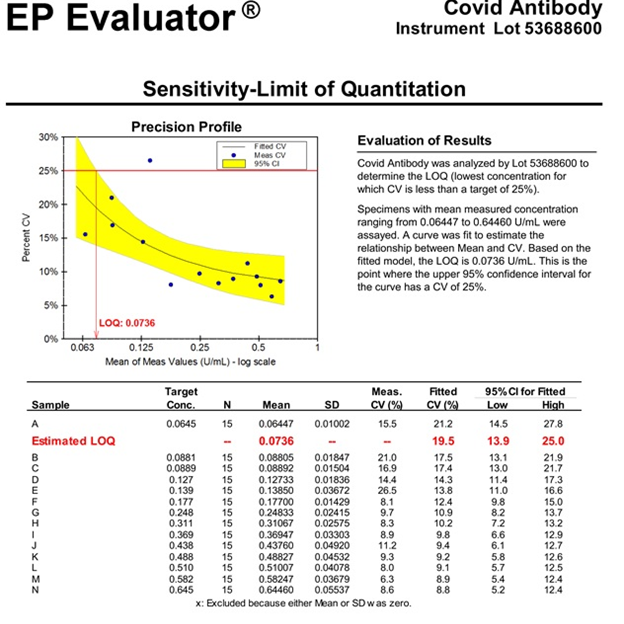


Table 7: DBS Limit of quantitation bias results (reagent lot 53688600)

| Level | | LOQ Sample Results (U/mL) | | | | | | | | | | | | | | | | |
| --- | --- | --- | --- | --- | --- | --- | --- | --- | --- | --- | --- | --- | --- | --- | --- | --- | --- | --- |
|  |  | Day 1 | | | Day 2 | | | Day 3 | | | Day 4 | | | Day 5 | | | Linearity | |
|  |  | 1 | 2 | 3 | 4 | 5 | 6 | 7 | 8 | 9 | 10 | 11 | 12 | 13 | 14 | 15 | Target | Bias |
| A | 0.0% | 0.0712 | 0.0617 | 0.0608 | 0.0669 | 0.0776 | 0.0591 | 0.0500 | 0.0409 | 0.0629 | 0.0682 | 0.0771 | 0.0751 | 0.0723 | 0.0625 | 0.0608 | 0.0830 | NA |
| B | 2.5% | 0.091 | 0.0897 | 0.134 | 0.0769 | 0.0927 | 0.0939 | 0.0534 | 0.0639 | 0.0708 | 0.101 | 0.0898 | 0.100 | 0.0854 | 0.0950 | 0.0833 | 0.0969 | NA |
| C | 5.0% | 0.0903 | 0.104 | 0.0835 | 0.0959 | 0.118 | 0.104 | 0.0781 | 0.0597 | 0.064 | 0.0853 | 0.0849 | 0.101 | 0.0895 | 0.0849 | 0.0907 | 0.111 | NA |
| D | 7.5% | 0.125 | 0.109 | 0.148 | 0.153 | 0.136 | 0.112 | 0.114 | 0.110 | 0.0974 | 0.114 | 0.136 | 0.126 | 0.124 | 0.154 | 0.152 | 0.125 | NA |
| E | 10% | 0.136 | 0.118 | 0.132 | 0.132 | 0.134 | 0.127 | 0.139 | 0.0865 | 0.108 | 0.139 | 0.143 | 0.154 | 0.258 | 0.139 | 0.132 | 0.139 | 0.0% |
| F | 20% | 0.176 | 0.184 | 0.181 | 0.180 | 0.188 | 0.198 | 0.150 | 0.152 | 0.186 | 0.172 | 0.177 | 0.155 | 0.192 | 0.181 | 0.183 | 0.194 | -8.7% |
| G | 30% | 0.245 | 0.268 | 0.224 | 0.269 | 0.271 | 0.289 | 0.242 | 0.250 | 0.208 | 0.258 | 0.204 | 0.268 | 0.255 | 0.246 | 0.228 | 0.249 | -0.4% |
| H | 40% | 0.313 | 0.470 | 0.298 | 0.379 | 0.332 | 0.313 | 0.306 | 0.281 | 0.280 | 0.28 | 0.299 | 0.324 | 0.334 | 0.293 | 0.316 | 0.305 | 5.3% |
| I | 50% | 0.398 | 0.393 | 0.403 | 0.363 | 0.408 | 0.384 | 0.324 | 0.396 | 0.303 | 0.350 | 0.387 | 0.371 | 0.394 | 0.329 | 0.339 | 0.360 | 2.5% |
| J | 60% | 0.478 | 0.492 | 0.625 | 0.477 | 0.46 | 0.442 | 0.517 | 0.406 | 0.433 | 0.325 | 0.414 | 0.413 | 0.434 | 0.433 | 0.368 | 0.416 | 7.7% |
| K | 70% | 0.488 | 0.53 | 0.527 | 0.497 | 0.569 | 0.54 | 0.478 | 0.496 | 0.457 | 0.510 | 0.425 | 0.503 | 0.458 | 0.401 | 0.445 | 0.471 | 3.6% |
| L | 80% | 0.478 | 0.557 | 0.59 | 0.487 | 0.503 | 0.545 | 0.432 | 0.518 | 0.528 | 0.491 | 0.473 | 0.541 | 0.473 | 0.493 | 0.542 | 0.527 | -3.2% |
| M | 90% | 0.570 | 0.580 | 0.603 | 0.586 | 0.615 | 0.632 | 0.617 | 0.543 | 0.514 | 0.598 | 0.611 | 0.625 | 0.535 | 0.562 | 0.543 | 0.582 | 0.0% |
| N | 100% | 0.731 | 0.674 | 0.641 | 0.637 | 0.687 | 0.652 | 0.600 | 0.633 | 0.684 | 0.649 | 0.702 | 0.677 | 0.559 | 0.629 | 0.514 | 0.638 | 1.1% |
|  |  | Levels E and M used to set Target Values | | | | | | | | | | | | | | |  |  |

# DBS Linearity

Linearity was performed using positive and negative serum pools that were admixed in 10% increments to create 11 different serum concentrations of SARS-CoV-2 antibodies. Contrived blood samples were created using these different admixtures and applied to DBS cards. Following extraction and measurement, the initial linearity results indicated a linearity from 0.0677 to 147 U/mL for DBS samples (Table 7). Additional linearity results indicated acceptable biases (≤ 20.0%) for all concentrations measured between 0.0974 and 323 U/mL for DBS samples (Table 8). DBS samples that initially measured greater than 250 U/mL were manually diluted 10-fold with Universal Diluent and re-measured.

Table 8: Initial DBS linearity results

| Linearity Sample Results (U/mL) | | | | | | | |
| --- | --- | --- | --- | --- | --- | --- | --- |
| Level | % | Rep. 1 | Rep. 2 | Rep. 3 | Mean | Target | Bias |
| A | 0.0% | 0.0490 | 0.0833 | 0.0677 | 0.0667 | 0.0667 | NA |
| B | 10% | 15.3 | 16.3 | 15.5 | 15.7 | 13.4 | 4.4% |
| C | 20% | 27.6 | 32.7 | 30.2 | 30.2 | 26.6 | 3.9% |
| D | 30% | 42.2 | 43.8 | 46.8 | 44.3 | 39.9 | 2.9% |
| E | 40% | 56.7 | 56.8 | 56.9 | 56.8 | 53.2 | -0.3% |
| F | 50% | 73.6 | 66.0 | 71.8 | 70.5 | 66.5 | -0.7% |
| G | 60% | 82.8 | 83.6 | 89.9 | 85.4 | 79.8 | 0.6% |
| H | 70% | 98.0 | 99.2 | 91.3 | 96.2 | 93.0 | -2.8% |
| I | 80% | 115 | 111 | 118 | 115 | 106 | 1.5% |
| J | 90% | 119 | 120 | 120 | 120 | 120 | -5.7% |
| K | 100% | 143 | 157 | 142 | 147 | 133 | 4.6% |
| Targets were set by linear regression using all levels. | | | | | | | |

Table 9: Additional DBS linearity results

| Linearity Sample Results (U/mL) | | | | | | | |
| --- | --- | --- | --- | --- | --- | --- | --- |
| Level | % | Rep. 1 | Rep. 2 | Rep. 3 | Mean | Target | Bias |
| A | 0% | 0.0840 | 0.0913 | 0.117 | 0.0974 | -3.07 | NA |
| B | 10% | 20.1 | 28.9 | 27.8 | 25.6 | 29.7 | -13.8% |
| C | 20% | 53.2 | 57.2 | 69.3 | 59.9 | 62.5 | -4.1% |
| D | 30% | 80.4 | 78.8 | 83.8 | 81 | 95.2 | -14.9% |
| E | 40% | 141 | 120 | 132 | 131 | 128 | 2.4% |
| F | 50% | 173 | 158 | 159 | 163 | 161 | 1.6% |
| G | 60% | 205 | 238 | 212 | 218 | 194 | 12.8% |
| H | 70% | 249 | 258* | 237 | 248 | 226 | 9.6% |
| I | 80% | 198 | 243 | 225 | 222 | 259 | -14.3% |
| J | 90% | 291* | 303* | 293* | 296 | 292 | 1.3% |
| K | 100% | 292* | 300* | 378* | 323 | 325 | -0.4% |

Targets were set by linear regression using all levels.

*Extracts were manually diluted 10-fold and re-measured. Results were multiplied by 10.

# Clinical Cutoff

In order to report DBS samples categorically negative or positive, a clinical cutoff value was determined. This was calculated using the self-collected DBS samples from donors that were confirmed to be seronegative and did not have an active COVID-19 infection (Donors with “B” pre-fix in Table 11). The mean and standard deviation for results from self-collected DBS samples were found to be 0.0625 and 0.0405 U/mL, respectively (n = 78). The cutoff was set by multiplying the standard deviation by three and adding the mean to give a value of 0.184 U/mL. As such, the clinical cutoff was set to 0.185 U/mL where all results greater than or equal to this value were considered positive (P) and all values less than 0.185 considered negative (N).

# DBS Inter-Assay Imprecision

Inter-assay imprecision was performed over 4 different days with two replicates being measured per run and two runs occurring per day for a total of 16 replicates from 6 different contrived blood samples. A single instrument was used for this study. DBS samples spanned an antibody concentration range of 0.517 (~3 times DBS cutoff) to 177 U/mL (~1000 times DBS cutoff) using two different reagent lots (53688601 and 54682501). Repeatability, within-laboratory imprecision, and total variance (% CV) was less than 20.0% (Table 9 and Table 10) for both reagent lots and is considered acceptable relative to FDA guidance for ligand binding assays (2). All the samples had total categorical agreement of 100.0%.

Table 10: Inter-assay imprecision of extracted DBS samples (reagent lot 53688601)

| Batch # (Date) | Replicate | Imprecision DBS Sample Results (U/mL) | | | | | | | | | | | |
| --- | --- | --- | --- | --- | --- | --- | --- | --- | --- | --- | --- | --- | --- |
|  |  | 1 | | 2 | | 3 | | 4 | | 5 | | 6 | |
|  |  | U/mL | Result | U/mL | Result | U/mL | Result | U/mL | Result | U/mL | Result | U/mL | Result |
| Day 1, Run 1 | 1 | 0.738 | P | 0.550 | P | 4.20 | P | 14.9 | P | 64.5 | P | 144 | P |
|  | 2 | 0.600 | P | 0.505 | P | 4.93 | P | 11.3 | P | 44.6 | P | 167 | P |
| Day 1, Run 2 | 3 | 0.664 | P | 0.573 | P | 5.57 | P | 16.3 | P | 60.0 | P | 151 | P |
|  | 4 | 0.722 | P | 0.568 | P | 5.97 | P | 15.8 | P | 70.2 | P | 149 | P |
| Day 2, Run 1 | 5 | 0.784 | P | 0.595 | P | 5.74 | P | 14.9 | P | 67.8 | P | 152 | P |
|  | 6 | 0.704 | P | 0.512 | P | 6.20 | P | 15.7 | P | 55.6 | P | 150 | P |
| Day 2, Run 2 | 7 | 0.702 | P | 0.577 | P | 5.74 | P | 16.1 | P | 70.9 | P | 169 | P |
|  | 8 | 0.754 | P | 0.538 | P | 6.48 | P | 15.6 | P | 70.0 | P | 165 | P |
| Day 3, Run 1 | 9 | 0.682 | P | 0.514 | P | 5.61 | P | 16.4 | P | 65.3 | P | 145 | P |
|  | 10 | 0.634 | P | 0.473 | P | 6.19 | P | 15.1 | P | 56.5 | P | 134 | P |
| Day 3, Run 2 | 11 | 0.632 | P | 0.581 | P | 6.33 | P | 15.3 | P | 57.5 | P | 151 | P |
|  | 12 | 0.765 | P | 0.580 | P | 6.37 | P | 16.4 | P | 62.8 | P | 177 | P |
| Day 4, Run 1 | 13 | 0.659 | P | 0.531 | P | 5.66 | P | 14.7 | P | 63.8 | P | 171 | P |
|  | 14 | 0.612 | P | 0.416 | P | 6.42 | P | 15.3 | P | 51.5 | P | 141 | P |
| Day 4, Run 2 | 15 | 0.611 | P | 0.449 | P | 6.95 | P | 16.2 | P | 66.5 | P | 154 | P |
|  | 16 | 0.645 | P | 0.511 | P | 7.13 | P | 15.1 | P | 61.7 | P | 168 | P |
| Total Mean | | 0.682 |  | 0.530 |  | 6.0 |  | 15.3 |  | 61.8 |  | 156 |  |
| Total SD | | 0.0588 |  | 0.0514 |  | 0.724 |  | 1.21 |  | 7.27 |  | 12.4 |  |
| Total CV | | 8.6% |  | 9.7% |  | 12.1% |  | 7.9% |  | 11.8% |  | 8.0% |  |
| Repeatability (Error) | SD | 0.0580 |  | 0.0399 |  | 0.459 |  | 1.05 |  | 7.70 |  | 11.9 |  |
|  | CV | 8.5% |  | 7.5% |  | 7.7% |  | 6.9% |  | 12.5% |  | 7.7% |  |
| Within-Laboratory Precision | SD | 0.0770 |  | 0.0577 |  | 0.878 |  | 1.32 |  | 7.70 |  | 13.6 |  |
|  | CV | 11.3% |  | 10.9% |  | 14.7% |  | 8.6% |  | 12.5% |  | 8.7% |  |
| Total Agreement (%) | |  | 100% |  | 100% |  | 100% |  | 100% |  | 100% |  | 100% |

N – negative, P – positive, A – results agree, D – results disagree

Table 11: Inter-Assay imprecision of extracted DBS samples (reagent lot 54862501)

| Batch # (Date) | Replicate | Imprecision DBS Sample Results (U/mL) | | | | | | | | | | | |
| --- | --- | --- | --- | --- | --- | --- | --- | --- | --- | --- | --- | --- | --- |
|  |  | 1 | | 2 | | 3 | | 4 | | 5 | | 6 | |
|  |  | U/mL | Result | U/mL | Result | U/mL | Result | U/mL | Result | U/mL | Result | U/mL | Result |
| Day 1, Run 1 | 1 | 0.725 | P | 0.522 | P | 4.24 | P | 14.7 | P | 65.7 | P | 145 | P |
|  | 2 | 0.587 | P | 0.494 | P | 4.88 | P | 11.4 | P | 43.8 | P | 168 | P |
| Day 1, Run 2 | 3 | 0.663 | P | 0.531 | P | 5.52 | P | 16.2 | P | 60.3 | P | 153 | P |
|  | 4 | 0.702 | P | 0.560 | P | 5.83 | P | 15.6 | P | 69.2 | P | 146 | P |
| Day 2, Run 1 | 5 | 0.740 | P | 0.549 | P | 5.74 | P | 15.3 | P | 69.3 | P | 154 | P |
|  | 6 | 0.712 | P | 0.525 | P | 6.10 | P | 15.4 | P | 55.2 | P | 155 | P |
| Day 2, Run 2 | 7 | 0.696 | P | 0.543 | P | 5.73 | P | 16.0 | P | 71.5 | P | 171 | P |
|  | 8 | 0.709 | P | 0.493 | P | 6.32 | P | 15.2 | P | 67.8 | P | 160 | P |
| Day 3, Run 1 | 9 | 0.660 | P | 0.492 | P | 5.45 | P | 16.4 | P | 65.5 | P | 152 | P |
|  | 10 | 0.658 | P | 0.476 | P | 6.52 | P | 15.6 | P | 57.2 | P | 137 | P |
| Day 3, Run 2 | 11 | 0.592 | P | 0.538 | P | 6.17 | P | 15.1 | P | 56.7 | P | 155 | P |
|  | 12 | 0.713 | P | 0.545 | P | 6.22 | P | 16.1 | P | 61.2 | P | 173 | P |
| Day 4, Run 1 | 13 | 0.651 | P | 0.512 | P | 5.79 | P | 14.8 | P | 65.7 | P | 173 | P |
|  | 14 | 0.592 | P | 0.415 | P | 6.40 | P | 15.4 | P | 52.4 | P | 139 | P |
| Day 4, Run 2 | 15 | 0.557 | P | 0.410 | P | 6.89 | P | 16.1 | P | 67.0 | P | 155 | P |
|  | 16 | 0.616 | P | 0.457 | P | 7.03 | P | 14.8 | P | 61.2 | P | 164 | P |
| Total Mean | | 0.661 |  | 0.504 |  | 5.93 |  | 15.3 |  | 61.9 |  | 156 |  |
| Total SD | | 0.0570 |  | 0.0456 |  | 0.706 |  | 1.16 |  | 7.39 |  | 11.3 |  |
| Total CV | | 8.6% |  | 9.1% |  | 11.9% |  | 7.6% |  | 11.9% |  | 7.3% |  |
| Repeatability (Error) | SD | 0.0505 |  | 0.0309 |  | 0.451 |  | 0.936 |  | 8.15 |  | 12.3 |  |
|  | CV | 7.6% |  | 6.1% |  | 7.6% |  | 6.1% |  | 13.2% |  | 7.9% |  |
| Within-Laboratory Precision | SD | 0.0668 |  | 0.0475 |  | 0.760 |  | 1.22 |  | 8.15 |  | 12.3 |  |
|  | CV | 10.1% |  | 9.4% |  | 12.8% |  | 8.0% |  | 13.2% |  | 7.9% |  |
| Total Agreement (%) | |  | 100% |  | 100% |  | 100% |  | 100% |  | 100% |  | 100% |

N – negative, P – positive, A – results agree, D – results disagree

# Clinical Performance Study

For the clinical performance studies, some results were not obtained for all sample types from all donors (Table 11). Either a sample was not provided for testing or the sample did not have sufficient volume for measurement (*e.g.* insufficient volume on DBS card for extraction and measurement, QNS). Serum samples that initially measured greater than 250 U/mL were manually diluted 10-fold with the approved Roche Universal Diluent and re-measured; results were multiplied by 10. In addition, some samples had results that were reported by the instrument as “< Test” indicating the signal generated was less than the intercept of the calibration curve used to calculate a concentration. Donors with a pre-fix of “A” previously tested positive for COVID-19 using an RT-PCR test while donors with a pre-fix of “B” are presumed negative.

All serum and DBS results where a definitive number was not obtained were excluded from quantitative analysis. Six donors (B10, B35, B51, B64, B-077, B80) that were reported to have never had COVID-19 as well as confirmed not to have an active SARS-CoV-2 infection had venous serum, professionally collected DBS, and self-collected DBS samples results all measure as positive. To confirm these results, serum was measured using three additional EUA approved antibody assay: Roche Elecsys Anti-SARS-CoV-2 (qualitative nucleocapsid assay), DiaSorin Liason SARS-CoV-2 S1/S2 IgG (qualitative spike protein assay) and DiaSorin Laison SARS-CoV-2 IgM (qualitative spike protein assay) (3). All six donors had a positive serum test result for at least one additional assay (Table 12). As such, these donors were excluded from qualitative and quantitative method comparison data analysis.

Table 12: Clinical performance results

|  | Self-Collection Experience | RT-PCR Results | Serum | | Self-Collection DBS | | Phlebotomist Collection DBS | |
| --- | --- | --- | --- | --- | --- | --- | --- | --- |
| ID | Result | Result | U/mL | Result | U/mL | Result | U/mL | Result |
| A1 | Yes | P | 866 | P | 53.5 | P | 62.6 | P |
| A2 | Yes | P | 1020 | P | 79 | P | 77.8 | P |
| A3 | Yes | P | 101 | P | 9.11 | P | 9.38 | P |
| A4 | Yes | P | 229 | P | 18.9 | P | 21.4 | P |
| A5 | Yes | P | 631 | P | 38.7 | P | QNS | NA |
| A6 | Yes | P | 2060 | P | 119 | P | 122 | P |
| A7 | Yes | P | 799 | P | 46.3 | P | 32.6 | P |
| A8 | Yes | P | 715 | P | > 250 | P | 53.6 | P |
| A9 | Yes | P | 1190 | P | 62.4 | P | 90.8 | P |
| A10 | Yes | P | > 2500 | P | > 250 | P | 222 | P |
| A11 | Yes | P | 2270 | P | 193 | P | 168 | P |
| A12 | Yes | P | 1230 | P | 67.1 | P | 103 | P |
| A13 | Yes | P | 60.8 | P | 4.26 | P | 4.27 | P |
| A14 | Yes | P | 533 | P | 35.0 | P | 32.7 | P |
| A15 | No | P | 18.4 | P | 1.77 | P | 1.54 | P |
| A16 | No | P | 242 | P | 18.1 | P | 19.5 | P |
| A17 | Yes | P | 281 | P | 74.6 | P | 17.6 | P |
| A18 | No | P | 506 | P | 35.6 | P | 36.4 | P |
| A19 | No | P | 602 | P | 42.8 | P | 41.5 | P |
| A20 | No | P | 0.0250 | N | 0.0416 | N | 0.0370 | N |
| A21 | No | P | 240 | P | 13.4 | P | 11.4 | P |
| A22 | No | P | > 2500 | P | > 250 | P | > 250 | P |
| A23 | No | P | 4.64 | P | 0.510 | P | 0.632 | P |
| A24 | No | P | 6.11 | P | 0.478 | P | 0.823 | P |
| A25 | No | P | 134 | P | QNS | NA | QNS | NA |
| A26 | No | P | 223 | P | 13.1 | P | 16.0 | P |
| A27 | No | P | Samples not received | | 1.63 | P | Samples not received | |
| A28 | No | P | 54.1 | P | Sample not collected | | 0.988 | P |
| A29 | No | P | 2.54 | P | 0.188 | P | 0.192 | P |
| A30 | No | P | 37.6 | P | 2.35 | P | 2.17 | P |
| A31 | No | P | 82.1 | P | 3.83 | P | 5.41 | P |
| A32 | No | P | 11.2 | P | 0.829 | P | 0.748 | P |
| A33 | No | P | 59.0 | P | 1.63 | P | 1.49 | P |
| A34 | No | P | 248 | P | 17.8 | P | 18.4 | P |
| A35 | Yes | P | 340 | P | 28.5 | P | 22.2 | P |
| A36 | No | P | 39.5 | P | 3.07 | P | 2.82 | P |

P – positive, N – negative, QNS – quantity not sufficient for measurement, < Test – below analytical measurement range

*Negative donors measured positive for serum, self-collection DBS, and professional collection DBS

|  | Self-Collection Experience | RT-PCR Results | Serum | | Self-Collection DBS | | Phlebotomist Collection DBS | |
| --- | --- | --- | --- | --- | --- | --- | --- | --- |
| ID | Result | Result | U/mL | Result | U/mL | Result | U/mL | Result |
| B1 | No | N | 0.0218 | N | 0.0358 | N | 0.0355 | N |
| B2 | No | N | 0.0179 | N | 0.0379 | N | 0.0400 | N |
| B3 | No | N | 0.0388 | N | 0.0360 | N | 0.0126 | N |
| B4 | No | N | 0.0177 | N | 0.0471 | N | 0.0516 | N |
| B5 | No | N | 0.0206 | N | 0.0408 | N | 0.0315 | N |
| B6 | No | N | 0.0253 | N | 0.0566 | N | 0.0332 | N |
| B7 | No | N | 0.0168 | N | 0.0237 | N | 0.0243 | N |
| B8 | No | N | 0.00200 | N | 0.0247 | N | 0.0196 | N |
| B9 | Yes | N | 0.0245 | N | 0.0382 | N | 0.0368 | N |
| *B10 | No | N | 33.4 | P | 2.26 | P | 2.33 | P |
| B11 | Yes | N | 0.0174 | N | 0.0484 | N | 0.0614 | N |
| B12 | No | N | 0.0264 | N | 0.0324 | N | 0.0322 | N |
| B13 | No | N | 0.0215 | N | 0.0257 | N | 0.0392 | N |
| B14 | No | N | < Test | N | 0.0607 | N | 0.0280 | N |
| B15 | No | N | 0.0142 | N | 0.0305 | N | 0.0430 | N |
| B16 | No | N | 0.0377 | N | 0.0523 | N | 0.0237 | N |
| B17 | Yes | N | 0.0358 | N | 0.0255 | N | 0.0450 | N |
| B18 | No | N | 0.0263 | N | 0.0350 | N | 0.0346 | N |
| B19 | No | N | 0.0248 | N | 0.0391 | N | 0.0334 | N |
| B20 | No | N | 0.0235 | N | 0.0256 | N | 0.0228 | N |
| B21 | No | N | 0.0289 | N | 0.0204 | N | 0.0407 | N |
| B22 | No | N | 0.0547 | N | 0.0534 | N | 0.0409 | N |
| B23 | No | N | 0.0269 | N | 0.0265 | N | 0.0347 | N |
| B24 | No | N | 0.0116 | N | 0.131 | N | 0.0414 | N |
| B25 | Yes | N | 0.0317 | N | QNS | NA | 0.0108 | N |
| B26 | No | N | 0.0425 | N | 0.0482 | N | 0.0351 | N |
| B27 | No | N | 0.0343 | N | 0.161 | N | 0.0579 | N |
| B28 | Yes | N | 0.0176 | N | 0.0627 | N | 0.0360 | N |
| B29 | No | N | 0.0118 | N | 0.0230 | N | 0.0335 | N |
| B30 | No | N | < Test | N | 0.0520 | N | 0.0234 | N |
| B31 | No | N | 0.0314 | N | QNS | NA | 0.0238 | N |
| B32 | No | N | 0.0221 | N | 0.108 | N | 0.0344 | N |
| B33 | No | N | 0.0232 | N | 0.0909 | N | 0.0308 | N |
| B34 | Yes | N | 0.0362 | N | 0.0331 | N | 0.0314 | N |
| *B35 | No | N | 359 | P | 21.8 | P | 22.8 | P |
| B36 | No | N | 0.0193 | N | 0.0399 | N | 0.0297 | N |
| B37 | Yes | N | 0.0489 | N | 0.0208 | N | 0.0209 | N |
| B38 | No | N | 0.0290 | N | 0.0540 | N | 0.0325 | N |
| B39 | Yes | N | 0.0183 | N | 0.121 | N | 0.0245 | N |
| B40 | No | N | 0.0162 | N | 0.0756 | N | 0.0319 | N |
| B41 | No | N | 0.0277 | N | 0.0454 | N | 0.0399 | N |
| B42 | No | N | 0.0193 | N | 0.0620 | N | 0.0336 | N |

P – positive, N – negative, QNS – quantity not sufficient for measurement, < Test – below analytical measurement range

*Negative donors measured positive for serum, self-collection DBS, and professional collection DBS

|  | Self-Collection Experience | RT-PCR Results | Serum | | Self-Collection DBS | | Phlebotomist Collection DBS | |
| --- | --- | --- | --- | --- | --- | --- | --- | --- |
| ID | Result | Result | U/mL | Result | U/mL | Result | U/mL | Result |
| B43 | No | N | 0.0214 | N | 0.105 | N | 0.0246 | N |
| B44 | No | N | 0.0245 | N | 0.0531 | N | 0.0278 | N |
| B45 | No | N | 0.0337 | N | 0.0530 | N | 0.0342 | N |
| B46 | No | N | 0.0141 | N | 0.0669 | N | 0.0351 | N |
| B47 | No | N | 0.0532 | N | 0.200 | P | 0.0324 | N |
| B48 | No | N | 0.00599 | N | 0.0724 | N | 0.0301 | N |
| B49 | Yes | N | 0.0246 | N | 0.0476 | N | 0.0303 | N |
| B50 | Yes | N | 0.015 | N | 0.0322 | N | 0.0226 | N |
| *B51 | Yes | N | 8.81 | P | 0.785 | P | 0.604 | P |
| B52 | No | N | 0.0254 | N | 0.245 | P | 0.0372 | N |
| B53 | Yes | N | 0.0316 | N | 0.120 | N | 0.0299 | N |
| B54 | Yes | N | 0.00622 | N | 0.0829 | N | 0.0383 | N |
| B55 | No | N | 0.0382 | N | 0.0364 | N | 0.0482 | N |
| B56 | No | N | 0.0261 | N | 0.0325 | N | 0.0484 | N |
| B57 | No | N | 0.0362 | N | 0.0462 | N | 0.0375 | N |
| B58 | No | N | 0.0347 | N | QNS | NA | 0.0302 | N |
| B59 | No | N | < Test | N | 0.0930 | N | 0.0338 | N |
| B60 | No | N | 0.0322 | N | 0.0337 | N | 0.00399 | N |
| B61 | No | N | 0.0272 | N | 0.0384 | N | 0.0371 | N |
| B62 | No | N | 0.0183 | N | 0.0298 | N | 0.0310 | N |
| B63 | Yes | N | 0.0378 | N | 0.0816 | N | 0.0404 | N |
| *B64 | No | N | 43.9 | P | 1.58 | P | 1.47 | P |
| B65 | No | N | 0.0188 | N | 0.0724 | N | 0.0834 | N |
| B66 | No | N | 0.0234 | N | 0.0452 | N | 0.0181 | N |
| B67 | Yes | N | 0.00853 | N | 0.0584 | N | 0.0376 | N |
| B68 | Yes | N | 0.0346 | N | 0.0995 | N | 0.0568 | N |
| B69 | No | N | 0.00904 | N | 0.0731 | N | 0.0128 | N |
| B70 | No | N | 0.0184 | N | 0.0811 | N | 0.0369 | N |
| B71 | No | N | 0.0357 | N | 0.0414 | N | 0.0324 | N |
| B72 | No | N | 0.0592 | N | 0.0483 | N | 0.0422 | N |
| B73 | No | N | 0.0204 | N | 0.0540 | N | 0.0921 | N |
| B74 | Yes | N | 0.0322 | N | 0.0661 | N | 0.0399 | N |
| B75 | Yes | N | 0.0352 | N | 0.0216 | N | 0.0120 | N |
| B76 | No | N | 0.0329 | N | 0.0976 | N | 0.0411 | N |
| *B77 | Yes | N | 534 | P | 43.4 | P | 39.0 | P |
| B78 | No | N | 0.0300 | N | 0.0555 | N | 0.0270 | N |
| B79 | Yes | N | 0.0101 | N | 0.0589 | N | 0.0330 | N |
| *B80 | Yes | N | 1170 | P | 80.3 | P | 70.6 | P |
| B81 | Yes | N | 0.00283 | N | 0.146 | N | 0.0464 | N |
| B82 | No | N | 0.00954 | N | 0.0512 | N | 0.0290 | N |
| B83 | No | N | 0.00606 | N | 0.0794 | N | 0.0243 | N |
| B84 | No | N | 0.0383 | N | 0.119 | N | 0.0144 | N |

P – positive, N – negative, QNS – quantity not sufficient for measurement, < Test – below analytical measurement range

*Negative donors measured positive for serum, self-collection DBS, and professional collection DBS

Table 13: Six “false” positive donor serum results from four EUA approved serological assays

|  |  | EUA Approved Assay | | | | | | | |
| --- | --- | --- | --- | --- | --- | --- | --- | --- | --- |
|  |  | Roche Elecsys Anti-SARS-CoV-2 S | | Roche Elecsys Anti-SARS-CoV-2 | | DiaSorin Liaison SARS-CoV-2 S1/S2 IgG | | DiaSorin Liaison SARS-CoV-2 IgM | |
|  | Target | Spike Protein | | Nucleocapsid | | Spike Protein | | Spike Protein | |
|  | Assay Type | Semi-Quantitative | | Qualitative | | Qualitative | | Qualitative | |
|  | Negative Range | < 0.8 U/mL | | < 1.0 COI | | < 12 AU/mL | | < 1.1 index | |
|  | Equivocal Range | not applicable | | not applicable | | 12 to 15 AU/mL | | not applicable | |
|  | Positive Range | ≥ 0.8 U/mL | | ≥ 1.0 COI | | ≥ 15 AU/mL | | ≥ 1.1 index | |
|  |  | Result (U/mL) | Interpret. | Result (COI) | Interpret. | Result (AU/mL) | Interpret. | Result (index) | Interpret. |
| Donor ID | B10 | 33.4 | P | 10.85 | P | 10.5 | N | 0.452 | N |
|  | B35 | 359 | P | 55.63 | P | 48 | P | 0.15 | N |
|  | B51 | 8.81 | P | 0.625 | N | 8.22 | N | 1.49 | P |
|  | B64 | 43.9 | P | 125.7 | P | 50.6 | P | 0.905 | N |
|  | B77 | 534 | P | 10.2 | P | 111 | P | 0.39 | N |
|  | B80 | 1170 | P | 0.089 | N | 233 | P | 0.132 | N |

# DBS Shipping Stability

Studies were performed to evaluate sample stability during the shipping process including storage of samples before shipping, samples sitting prior to pick up, and conditions following pick up and transport to the lab for testing (4,5). Contrived blood samples (n = 76) were prepared with antibody concentrations ranging from 0.0358 U/mL (~0.2 times DBS cutoff) to 4.91 U/mL (~27 times DBS cutoff); most samples (n = 70) were within 5 times the DBS cutoff. Samples were made into triplicates and were divided into three shipping conditions: ambient (20-25°C), winter, and summer (refer to Table 13). Samples were measured in parallel whereas the room temperature samples were used as baseline results. Results that were less than the assay’s DBS LOQ were excluded from bias analysis but included in qualitative analysis. Winter and summer excursion samples had a total categorical agreement of 97.4% and mean biases of 6.0% and -0.5%, respectively. One winter excursion sample that did not have categorical agreement had a baseline concentration of 0.0499 U/mL and a concentration post-excursion of 0.269 U/mL which is within 1.5 times the DBS clinical cutoff (0.185 U/mL). The other winter-excursion sample that did not have categorical agreement with baseline results had a baseline concentration of 0.185 U/mL which is equivalent to the DBS clinical cutoff (0.185 U/mL). The two summer-excursion samples that did not have categorical agreement with baseline results included this sample with baseline results equivalent to the DBS clinical cutoff and a sample with baseline results of 0.233 U/mL which is within 26% of the DBS clinical cutoff.

Table 14: Simulated shipping conditions

| **Cycle Period** | **Winter Temperatures*** | **Summer Temperatures*** | **Hours** |
| --- | --- | --- | --- |
| **1** | 22°C (drying following creation) | | 3 |
| **2** | 22°C (storage in plastic specimen pouch) | | 5 |
| **3** | -20°C | 40°C | 8 |
| **4** | 22°C | 22°C | 4 |
| **5** | -20°C | 40°C | 2 |
| **6** | 4°C | 30°C | 36 |
| **7** | -20°C | 40°C | 6 |
| *within ±2°C | | | |

Table 15: DBS shipping excursion results

| Sample | Ambient | | Winter Excursion | | | | Summer Excursion | | | |
| --- | --- | --- | --- | --- | --- | --- | --- | --- | --- | --- |
|  | U/mL | Result | U/mL | Bias | Result | Agree / Disagree | U/mL | Bias | Result | Agree / Disagree |
| 1 | 0.0358 | N | 0.0624 | NA | N | A | 0.140 | NA | N | A |
| 2 | 0.0446 | N | 0.119 | NA | N | A | 0.0612 | NA | N | A |
| 3 | 0.0455 | N | 0.0469 | NA | N | A | 0.0650 | NA | N | A |
| 4 | 0.0499 | N | 0.269 | NA | P | D | 0.0978 | NA | N | A |
| 5 | 0.0534 | N | 0.102 | NA | N | A | 0.0681 | NA | N | A |
| 6 | 0.0641 | N | 0.0370 | NA | N | A | 0.0288 | NA | N | A |
| 7 | 0.0663 | N | 0.0384 | NA | N | A | 0.0473 | NA | N | A |
| 8 | 0.0800 | N | 0.0390 | NA | N | A | 0.0434 | NA | N | A |
| 9 | 0.0966 | N | 0.0398 | NA | N | A | 0.0232 | NA | N | A |
| 10 | 0.111 | N | 0.0405 | NA | N | A | 0.0281 | NA | N | A |
| 11 | 0.113 | N | 0.109 | NA | N | A | 0.112 | NA | N | A |
| 12 | 0.123 | N | 0.137 | NA | N | A | 0.148 | NA | N | A |
| 13 | 0.151 | N | 0.171 | NA | N | A | 0.142 | NA | N | A |
| 14 | 0.164 | N | 0.183 | NA | N | A | 0.121 | NA | N | A |
| 15 | 0.169 | N | 0.173 | NA | N | A | 0.177 | NA | N | A |
| 16 | 0.176 | N | 0.155 | NA | N | A | 0.138 | NA | N | A |
| 17 | 0.177 | N | 0.181 | NA | N | A | 0.147 | NA | N | A |
| 18 | 0.185 | P | 0.174 | NA | N | D | 0.144 | NA | N | D |
| 19 | 0.195 | P | 0.497 | 154.9% | P | A | 0.242 | 24.1% | P | A |
| 20 | 0.205 | P | 0.237 | 15.6% | P | A | 0.220 | 7.3% | P | A |
| 21 | 0.205 | P | 0.232 | 13.2% | P | A | 0.220 | 7.3% | P | A |
| 22 | 0.212 | P | 0.218 | 2.8% | P | A | 0.236 | 11.3% | P | A |
| 23 | 0.233 | P | 0.209 | -10.3% | P | A | 0.165 | NA | N | D |
| 24 | 0.250 | P | 0.206 | -17.6% | P | A | 0.194 | -22.4% | P | A |
| 25 | 0.259 | P | 0.235 | -9.3% | P | A | 0.235 | -9.3% | P | A |
| 26 | 0.259 | P | 0.221 | -14.7% | P | A | 0.196 | -24.3% | P | A |
| 27 | 0.262 | P | 0.223 | -14.9% | P | A | 0.242 | -7.6% | P | A |
| 28 | 0.277 | P | 0.259 | -6.5% | P | A | 0.230 | -17.0% | P | A |
| 29 | 0.304 | P | 0.242 | -20.4% | P | A | 0.284 | -6.6% | P | A |
| 30 | 0.322 | P | 0.248 | -23.0% | P | A | 0.318 | -1.2% | P | A |
| 31 | 0.330 | P | 0.385 | 16.7% | P | A | 0.318 | -3.6% | P | A |
| 32 | 0.332 | P | 0.362 | 9.0% | P | A | 0.328 | -1.2% | P | A |
| 33 | 0.334 | P | 0.322 | -3.6% | P | A | 0.360 | 7.8% | P | A |
| 34 | 0.344 | P | 0.422 | 22.7% | P | A | 0.362 | 5.2% | P | A |
| 35 | 0.357 | P | 0.409 | 14.6% | P | A | 0.352 | -1.4% | P | A |
| 36 | 0.359 | P | 0.349 | -2.8% | P | A | 0.386 | 7.5% | P | A |
| 37 | 0.360 | P | 0.458 | 27.2% | P | A | 0.369 | 2.5% | P | A |
| 38 | 0.360 | P | 0.430 | 19.4% | P | A | 0.397 | 10.3% | P | A |

N – negative, P – positive, A – results agree, D – results disagree

| Sample | Ambient | | | Winter Excursion | | | | | | | Summer Excursion | | | | | | | |  |
| --- | --- | --- | --- | --- | --- | --- | --- | --- | --- | --- | --- | --- | --- | --- | --- | --- | --- | --- | --- |
|  | U/mL | Result | | U/mL | Bias | | Result | | Agree / Disagree | | U/mL | | Bias | | Result | | Agree / Disagree | |  |
| 39 | 0.365 | | P | 0.353 | | -3.3% | | P | | A | | 0.357 | | -2.2% | | P | | A | |
| 40 | 0.365 | | P | 0.473 | | 29.6% | | P | | A | | 0.401 | | 9.9% | | P | | A | |
| 41 | 0.366 | | P | 0.399 | | 9.0% | | P | | A | | 0.345 | | -5.7% | | P | | A | |
| 42 | 0.371 | | P | 0.393 | | 5.9% | | P | | A | | 0.376 | | 1.3% | | P | | A | |
| 43 | 0.372 | | P | 0.435 | | 16.9% | | P | | A | | 0.397 | | 6.7% | | P | | A | |
| 44 | 0.386 | | P | 0.439 | | 13.7% | | P | | A | | 0.424 | | 9.8% | | P | | A | |
| 45 | 0.394 | | P | 0.427 | | 8.4% | | P | | A | | 0.403 | | 2.3% | | P | | A | |
| 46 | 0.394 | | P | 0.425 | | 7.9% | | P | | A | | 0.404 | | 2.5% | | P | | A | |
| 47 | 0.394 | | P | 0.437 | | 10.9% | | P | | A | | 0.387 | | -1.8% | | P | | A | |
| 48 | 0.402 | | P | 0.416 | | 3.5% | | P | | A | | 0.405 | | 0.7% | | P | | A | |
| 49 | 0.405 | | P | 0.443 | | 9.4% | | P | | A | | 0.455 | | 12.3% | | P | | A | |
| 50 | 0.414 | | P | 0.496 | | 19.8% | | P | | A | | 0.445 | | 7.5% | | P | | A | |
| 51 | 0.422 | | P | 0.436 | | 3.3% | | P | | A | | 0.431 | | 2.1% | | P | | A | |
| 52 | 0.439 | | P | 0.439 | | 0.0% | | P | | A | | 0.436 | | -0.7% | | P | | A | |
| 53 | 0.456 | | P | 0.464 | | 1.8% | | P | | A | | 0.445 | | -2.4% | | P | | A | |
| 54 | 0.474 | | P | 0.492 | | 3.8% | | P | | A | | 0.441 | | -7.0% | | P | | A | |
| 55 | 0.486 | | P | 0.496 | | 2.1% | | P | | A | | 0.491 | | 1.0% | | P | | A | |
| 56 | 0.488 | | P | 0.619 | | 26.8% | | P | | A | | 0.529 | | 8.4% | | P | | A | |
| 57 | 0.499 | | P | 0.502 | | 0.6% | | P | | A | | 0.422 | | -15.4% | | P | | A | |
| 58 | 0.551 | | P | 0.532 | | -3.4% | | P | | A | | 0.588 | | 6.7% | | P | | A | |
| 59 | 0.672 | | P | 0.657 | | -2.2% | | P | | A | | 0.743 | | 10.6% | | P | | A | |
| 60 | 0.705 | | P | 0.736 | | 4.4% | | P | | A | | 0.718 | | 1.8% | | P | | A | |
| 61 | 0.714 | | P | 0.730 | | 2.8% | | P | | A | | 0.654 | | -8.4% | | P | | A | |
| 62 | 0.752 | | P | 0.759 | | 0.9% | | P | | A | | 0.641 | | -14.8% | | P | | A | |
| 63 | 0.811 | | P | 0.750 | | -7.5% | | P | | A | | 0.803 | | -1.0% | | P | | A | |
| 64 | 0.812 | | P | 0.929 | | 14.4% | | P | | A | | 0.822 | | 1.2% | | P | | A | |
| 65 | 0.900 | | P | 0.980 | | 8.9% | | P | | A | | 0.886 | | -1.6% | | P | | A | |
| 66 | 0.902 | | P | 0.937 | | 3.9% | | P | | A | | 0.893 | | -1.0% | | P | | A | |
| 67 | 0.981 | | P | 0.988 | | 0.7% | | P | | A | | 1.04 | | 6.0% | | P | | A | |
| 68 | 1.04 | | P | 0.930 | | -10.8% | | P | | A | | 0.954 | | -8.3% | | P | | A | |
| 69 | 1.05 | | P | 1.00 | | -4.8% | | P | | A | | 0.970 | | -7.6% | | P | | A | |
| 70 | 1.17 | | P | 1.10 | | -6.0% | | P | | A | | 1.23 | | 5.1% | | P | | A | |
| 71 | 1.28 | | P | 1.35 | | 5.5% | | P | | A | | 1.25 | | -2.3% | | P | | A | |
| 72 | 1.52 | | P | 1.60 | | 5.3% | | P | | A | | 1.57 | | 3.3% | | P | | A | |
| 73 | 1.53 | | P | 1.62 | | 5.9% | | P | | A | | 1.40 | | -8.5% | | P | | A | |
| 74 | 1.71 | | P | 1.61 | | -5.8% | | P | | A | | 1.15 | | -32.7% | | P | | A | |
| 75 | 2.31 | | P | 2.27 | | -1.7% | | P | | A | | 2.24 | | -3.0% | | P | | A | |
| 76 | 4.68 | | P | 4.30 | | -8.1% | | P | | A | | 4.91 | | 4.9% | | P | | A | |
|  |  | |  | Mean Bias: | | 6.0% | | Total Agreement: | | 97.4% | | Mean Bias | | -0.5% | | Total Agreement: | | 97.4% | |
|  |  | |  | n | | 58 | | n | | 76 | | n | | 57 | | n | | 76 | |

N – negative, P – positive, A – results agree, D – results disagree

# Robustness Studies

Additional robustness studies were performed to stress different aspects of sample collection. Contrived blood samples were prepared with antibody concentrations ranging from 0.0380 U/mL (~0.2 times DBS cutoff) to 1.52 U/mL (~8 times DBS cutoff); most samples were within 5 times the DBS cutoff. All samples used to generate baseline results were spotted and dried for three hours prior to storage; no interferents or contamination was introduced. For acceptance of results, biases were determined to baseline measurements for results greater than the assay’s LOQ. A mean bias of 20% was used as quantitative acceptance following FDA guidance for ligand binding assay (2). For qualitative assessment, a total categorical agreement of 95.0% was utilized based on guidance from the FDA’s Home Specimen Collection Serology Template (4).

## Alternate Drying Times

To test the effects of drying time on sample results, alternative drying times were investigated using antibody concentrations designed to stress the clinical cutoff. Drying times prior to storage were 0, 1, 3 (baseline), and 22 hours (overnight). Following the pre-determined drying time, samples were placed in plastic specimen pouches (without desiccant) and stored at room temperature until measurement. DBS samples that were immediately stored after being spotted demonstrated total categorical agreement of 95.0% and mean bias of -32.5% and total categorical agreement of 95.0% versus DBS samples stored for 3 hours prior to placing in a desiccant free specimen pouch. The one sample that did not have categorical agreement with baseline results had a baseline concentration of 0.193 U/mL which is within 5% of the DBS clinical cutoff (0.185 U/mL). DBS samples that dried for one hour demonstrated total categorical agreement of 95.0% and mean bias of 1.3%. The one sample that did not have categorical agreement with baseline results had a baseline concentration of 0.176 U/mL which is again within 5% of the DBS clinical cutoff (0.185 U/mL). DBS samples stored for 22 hours demonstrated total categorical agreement of 100% and mean bias of 1.3% (Table 15). These results indicate that at least 1 hour of drying is required prior to sample storage and shipment. Risk of donors not allowing sufficient drying can be mitigated by appropriate design of donor materials including instructions for use (IFUs) and demonstration videos.

Table 16: Alternative drying times of 0, 1, 3 (baseline) and 22 hours for DBS samples

| Sample | Baseline (3 Hrs) | | Drying Time Prior to Storage (Hrs) | | | | | | | | | | | |
| --- | --- | --- | --- | --- | --- | --- | --- | --- | --- | --- | --- | --- | --- | --- |
|  |  |  | 0 Hrs | | | | 1 Hr | | | | 22 Hrs | | | |
|  | U/mL | Result | U/mL | Bias | Result | Agree/ Disagree | U/mL | Bias | Result | Agree/ Disagree | U/mL | Bias | Result | Agree/ Disagree |
| 1 | 0.0380 | N | 0.0594 | NA | N | A | 0.0686 | NA | N | A | 0.0553 | NA | N | A |
| 2 | 0.0439 | N | 0.0396 | NA | N | A | 0.0456 | NA | N | A | 0.0346 | NA | N | A |
| 3 | 0.0465 | N | 0.0770 | NA | N | A | 0.0668 | NA | N | A | 0.0255 | NA | N | A |
| 4 | 0.0512 | N | 0.0427 | NA | N | A | 0.0258 | NA | N | A | 0.0430 | NA | N | A |
| 5 | 0.0803 | N | 0.0491 | NA | N | A | 0.130 | NA | N | A | 0.0568 | NA | N | A |
| 6 | 0.166 | N | 0.144 | NA | N | A | 0.177 | NA | N | A | 0.170 | NA | N | A |
| 7 | 0.176 | N | 0.147 | NA | N | A | 0.233 | NA | P | D | 0.164 | NA | N | A |
| 8 | 0.193 | P | 0.136 | NA | N | D | 0.204 | 5.7% | P | A | 0.201 | 5.7% | P | A |
| 9 | 0.361 | P | 0.281 | -22.2% | P | A | 0.376 | 4.2% | P | A | 0.366 | 4.2% | P | A |
| 10 | 0.410 | P | 0.295 | -28.0% | P | A | 0.411 | 0.20% | P | A | 0.435 | 0.2% | P | A |
| 11 | 0.488 | P | 0.336 | -31.1% | P | A | 0.511 | 4.7% | P | A | 0.495 | 4.7% | P | A |
| 12 | 0.657 | P | 0.395 | -39.9% | P | A | 0.623 | -5.2% | P | A | 0.595 | -5.2% | P | A |
| 13 | 0.684 | P | 0.467 | -31.7% | P | A | 0.630 | -7.9% | P | A | 0.645 | -7.9% | P | A |
| 14 | 0.837 | P | 0.623 | -25.6% | P | A | 0.890 | 6.3% | P | A | 0.933 | 6.3% | P | A |
| 15 | 0.926 | P | 0.674 | -27.2% | P | A | 0.987 | 6.6% | P | A | 0.821 | 6.6% | P | A |
| 16 | 0.936 | P | 0.579 | -38.1% | P | A | 0.907 | -3.1% | P | A | 0.946 | -3.1% | P | A |
| 17 | 0.987 | P | 0.594 | -39.8% | P | A | 0.830 | -15.9% | P | A | 0.811 | -15.9% | P | A |
| 18 | 1.36 | P | 0.839 | -38.3% | P | A | 1.29 | -5.1% | P | A | 1.46 | -5.1% | P | A |
| 19 | 1.48 | P | 1.06 | -28.4% | P | A | 1.49 | 0.7% | P | A | 1.45 | 0.7% | P | A |
| 20 | 1.52 | P | 0.919 | -39.5% | P | A | 1.40 | -7.9% | P | A | 1.39 | -7.9% | P | A |
|  |  |  | Mean Bias | -32.5% | Total Agreement: | 95.0% | Mean Bias | 1.3% | Total Agreement: | 95.0% | Mean Bias | 1.3% | Total Agreement: | 100.0% |

N – negative, P – positive, A – results agree, D – results disagree

## DBS Card Drying in Humid Environment

Studies were performed to evaluate the effects of DBS samples drying under humid conditions. Four sample replicates were created for each concentration, where 3 of the 4 DBS samples were placed into an incubator at 40°C with a water pan in the bottom to simulate a relative humidity greater than 95%. The 4th sample replicate (baseline) was stored at room temperature and placed in a plastic specimen pouch after 3 hours of drying.

All samples that remained in a humid environment demonstrated total categorical agreement of 95.0%, with increasing mean biases of -13.3% (1 hour), -22.2% (3 hours) and -44.6% (22 hours, Table 16). Samples that did not have categorical agreement with baseline results had baseline concentrations within 11% of the DBS clinical cutoff (0.185 U/mL). These results indicate that prolonged drying (> 1 hour) in a humid environment may lead to unacceptable negative bias in sample results. Risk of donors drying samples inappropriately can be mitigated by appropriate design of donor materials including instructions for use (IFUs) and demonstration videos.

Table 17: Impact of humidity during drying for DBS samples for 1, 3 and 22 hours).

| Sample |  |  | Drying in Humid Environment Time Prior to Storage (Hrs) | | | | | | | | | | | |
| --- | --- | --- | --- | --- | --- | --- | --- | --- | --- | --- | --- | --- | --- | --- |
|  | Baseline (3 Hrs) | | 1 Hr | | | | 3 Hrs | | | | 22 Hrs | | | |
|  | U/mL | Result | U/mL | Bias | Result | Agree/ Disagree | U/mL | Bias | Result | Agree/ Disagree | U/mL | Bias | Result | Agree/ Disagree |
| 1 | 0.0380 | N | 0.0543 | NA | N | A | 0.0386 | NA | N | A | 0.137 | NA | N | A |
| 2 | 0.0439 | N | 0.0521 | NA | N | A | 0.0384 | NA | N | A | 0.0397 | NA | N | A |
| 3 | 0.0465 | N | 0.0936 | NA | N | A | 0.0632 | NA | N | A | 0.0768 | NA | N | A |
| 4 | 0.0512 | N | 0.0609 | NA | N | A | 0.0259 | NA | N | A | 0.0387 | NA | N | A |
| 5 | 0.0803 | N | 0.0375 | NA | N | A | 0.0330 | NA | N | A | 0.0806 | NA | N | A |
| 6 | 0.166 | N | 0.550 | NA | P | D | 0.175 | NA | N | A | 0.115 | NA | N | A |
| 7 | 0.176 | N | 0.165 | NA | N | A | 0.160 | NA | N | A | 0.0906 | NA | N | A |
| 8 | 0.193 | P | 0.186 | -3.6% | P | A | 0.151 | NA | N | D | 0.0814 | NA | N | D |
| 9 | 0.361 | P | 0.309 | -14.4% | P | A | 0.297 | -17.7% | P | A | 0.192 | -46.8% | P | A |
| 10 | 0.410 | P | 0.368 | -10.2% | P | A | 0.303 | -26.1% | P | A | 0.231 | -43.7% | P | A |
| 11 | 0.488 | P | 0.427 | -12.5% | P | A | 0.370 | -24.2% | P | A | 0.309 | -36.7% | P | A |
| 12 | 0.657 | P | 0.532 | -19.0% | P | A | 0.491 | -25.3% | P | A | 0.333 | -49.3% | P | A |
| 13 | 0.684 | P | 0.593 | -13.3% | P | A | 0.494 | -27.8% | P | A | 0.353 | -48.4% | P | A |
| 14 | 0.837 | P | 0.739 | -11.7% | P | A | 0.684 | -18.3% | P | A | 0.419 | -49.9% | P | A |
| 15 | 0.926 | P | 0.851 | -8.1% | P | A | 0.758 | -18.1% | P | A | 0.530 | -42.8% | P | A |
| 16 | 0.936 | P | 0.789 | -15.7% | P | A | 0.781 | -16.6% | P | A | 0.541 | -42.2% | P | A |
| 17 | 0.987 | P | 0.793 | -19.7% | P | A | 0.690 | -30.1% | P | A | 0.527 | -46.6% | P | A |
| 18 | 1.36 | P | 1.23 | -9.6% | P | A | 1.15 | -15.4% | P | A | 0.791 | -41.8% | P | A |
| 19 | 1.48 | P | 1.23 | -16.9% | P | A | 1.19 | -19.6% | P | A | 0.908 | -38.6% | P | A |
| 20 | 1.52 | P | 1.25 | -17.8% | P | A | 1.10 | -27.6% | P | A | 0.793 | -47.8% | P | A |
|  |  |  | Mean Bias | -13.3% | Total Agreement: | 95.0% | Mean Bias | -22.2% | Total Agreement: | 95.0% | Mean Bias | -44.6% | Total Agreement: | 95.0% |

N – negative, P – positive, A – results agree, D – results disagree

## Effects of Alcohol Exposure to DBS cards

The potential effects of alcohol exposure to DBS cards prior to addition of blood was investigated in the event that an individual did not allow their finger to dry after sterilization with an alcohol wipe. This study was performed by wiping an alcohol pad on a DBS card immediately prior to the addition of contrived blood samples to the card. For this study all samples were stored after three hours of drying and stored at room temperature. All samples demonstrated total categorical agreement of 100%, with a mean bias of -11.0% (Table 17). As this study represents a worst case scenario to alcohol exposure (saturation of DBS card with alcohol), these results are considered acceptable. Risk of donors exposing alcohol to blood can be mitigated by appropriate design of donor materials including instructions for use (IFUs) and demonstration videos.

Table 18: DBS alcohol exposure results

| Sample | Baseline | | Alcohol | | | |
| --- | --- | --- | --- | --- | --- | --- |
|  | U/mL | Result | U/mL | Bias | Result | Agree/ Disagree |
| 1 | 0.0380 | N | 0.138 | NA | N | A |
| 2 | 0.0439 | N | 0.0727 | NA | N | A |
| 3 | 0.0465 | N | 0.0943 | NA | N | A |
| 4 | 0.0512 | N | 0.0558 | NA | N | A |
| 5 | 0.0803 | N | 0.182 | NA | N | A |
| 6 | 0.166 | N | 0.174 | NA | N | A |
| 7 | 0.176 | N | 0.139 | NA | N | A |
| 8 | 0.193 | P | 0.220 | 14.0% | P | A |
| 9 | 0.361 | P | 0.360 | -0.3% | P | A |
| 10 | 0.410 | P | 0.348 | -15.1% | P | A |
| 11 | 0.488 | P | 0.411 | -15.8% | P | A |
| 12 | 0.657 | P | 0.431 | -34.4% | P | A |
| 13 | 0.684 | P | 0.833 | 21.8% | P | A |
| 14 | 0.837 | P | 0.754 | -9.9% | P | A |
| 15 | 0.926 | P | 0.855 | -7.7% | P | A |
| 16 | 0.936 | P | 0.812 | -13.2% | P | A |
| 17 | 0.987 | P | 0.808 | -18.1% | P | A |
| 18 | 1.36 | P | 1.11 | -18.4% | P | A |
| 19 | 1.48 | P | 1.15 | -22.3% | P | A |
| 20 | 1.52 | P | 1.17 | -23.0% | P | A |
|  |  |  | Mean Bias | -11.0% | Total Agreement: | 100.0% |

N – negative, P – positive, A – results agree, D – results disagree

## Effects of Finger Contamination

The effects of an unsterilized finger being exposed to a DBS spot was investigated by pressing an ungloved finger directly on a DBS card immediately prior to the addition of contrived blood samples. For this study all samples were stored after three hours of drying and stored at room temperature. Total qualitative categorical agreement of 95.0% was observed with mean bias of 1.3% (Table 18). As this study represents a worst case scenario to finger exposure, these results are considered acceptable. The one sample that did not have categorical agreement with baseline results had a baseline concentration of 0.208 U/mL which is within 13% of the DBS clinical cutoff (0.185 U/mL). Risk of donors contaminating DBS cards prior to addition of blood can be mitigated by appropriate design of donor materials including instructions for use (IFUs) and demonstration videos.

Table 19: DBS spot contamination with an unsterilized finger results

| Sample | Baseline | | Finger | | | |
| --- | --- | --- | --- | --- | --- | --- |
|  | U/mL | Result | U/mL | Bias | Result | Agree/ Disagree |
| 1 | 0.0342 | N | 0.0416 | NA | N | A |
| 2 | 0.0364 | N | 0.0444 | NA | N | A |
| 3 | 0.0382 | N | 0.0524 | NA | N | A |
| 4 | 0.0402 | N | 0.0372 | NA | N | A |
| 5 | 0.0450 | N | 0.0557 | NA | N | A |
| 6 | 0.208 | P | 0.175 | NA | N | D |
| 7 | 0.258 | P | 0.288 | 11.6% | P | A |
| 8 | 0.266 | P | 0.270 | 1.5% | P | A |
| 9 | 0.407 | P | 0.400 | -1.7% | P | A |
| 10 | 0.443 | P | 0.410 | -7.4% | P | A |
| 11 | 0.458 | P | 0.479 | 4.6% | P | A |
| 12 | 0.580 | P | 0.568 | -2.1% | P | A |
| 13 | 0.591 | P | 0.592 | 0.2% | P | A |
| 14 | 0.595 | P | 0.606 | 1.8% | P | A |
| 15 | 0.652 | P | 0.712 | 9.2% | P | A |
| 16 | 0.853 | P | 0.829 | -2.8% | P | A |
| 17 | 0.914 | P | 0.755 | -17.4% | P | A |
| 18 | 0.969 | P | 1.03 | 6.3% | P | A |
| 19 | 1.03 | P | 1.11 | 7.8% | P | A |
| 20 | 1.33 | P | 1.42 | 6.8% | P | A |
|  |  |  | Total Bias: | 1.3% | Total Agreement: | 95.0% |

N – negative, P – positive, A – results agree, D – results disagree

# Analytic Interference determination for DBS samples

Studies were also performed to test the effects of different interferences on the measurement of SARS-CoV-2 antibodies from DBS samples. Contrived blood samples were prepared with antibody concentrations ranging from 0.0342 U/mL (~0.2 times DBS cutoff) to 1.52 U/mL (~175 times DBS cutoff); most samples were within 5 times the DBS cutoff. All samples used to generate baseline results were spotted and dried for three hours prior to being stored. For acceptance of results, biases were determined to baseline measurements for results greater than the assay’s LOD. A mean bias of 20.0% was used as quantitative acceptance following FDA guidance for ligand binding assays (2). For acceptance of qualitative results, a total categorical agreement of 95.0% was utilized based on guidance from the FDA’s Home Specimen Collection Serology Template *(5)*.

## DBS Hemolysis Interference Study

To test the effects of hemolysis, contrived liquid blood samples were prepared in duplicate aliquots. One of the aliquots was frozen at -70°C for at least 30 minutes and thawed in order to lyse the red blood cells. Both aliquots (lysed and un-lysed) were then spotted onto DBS cards, dried and stored prior to testing. A total qualitative categorical agreement of 95.0% to un-lysed measurements was observed, one sample disagreed (D) with baseline results with a measured concentration of 0.208 U/mL, within 13% of the DBS clinical cutoff (0.185 U/mL). Lysed DBS samples demonstrated an acceptable mean bias of 3.6% compared to baseline measurements (Table 19).

Table 20: DBS hemolysis interference results

| Hemolysis Interference DBS results (U/mL) | | | | | | |
| --- | --- | --- | --- | --- | --- | --- |
| Sample | Baseline | | Hemolyzed | | | |
|  | U/mL | Result | U/mL | Bias | Result | Agree/ Disagree |
| 1 | 0.0342 | N | 0.0692 | NA | N | A |
| 2 | 0.0364 | N | 0.0774 | NA | N | A |
| 3 | 0.0382 | N | 0.0516 | NA | N | A |
| 4 | 0.0402 | N | 0.0658 | NA | N | A |
| 5 | 0.045 | N | 0.0538 | NA | N | A |
| 6 | 0.208 | P | 0.184 | -11.5% | N | D |
| 7 | 0.258 | P | 0.319 | 23.6% | P | A |
| 8 | 0.266 | P | 0.291 | 9.4% | P | A |
| 9 | 0.407 | P | 0.443 | 8.8% | P | A |
| 10 | 0.443 | P | 0.432 | -2.5% | P | A |
| 11 | 0.458 | P | 0.509 | 11.1% | P | A |
| 12 | 0.580 | P | 0.586 | 1.0% | P | A |
| 13 | 0.591 | P | 0.692 | 17.1% | P | A |
| 14 | 0.595 | P | 0.661 | 11.1% | P | A |
| 15 | 0.652 | P | 0.677 | 3.8% | P | A |
| 16 | 0.853 | P | 0.803 | -5.9% | P | A |
| 17 | 0.914 | P | 0.854 | -6.6% | P | A |
| 18 | 0.969 | P | 0.965 | -0.4% | P | A |
| 19 | 1.03 | P | 1.07 | 3.9% | P | A |
| 20 | 1.33 | P | 1.22 | -8.3% | P | A |
|  |  |  | Total Bias: | 3.6% | Total Agreement: | 95.0% |

N – negative, P – positive, A – results agree, D – results disagree

## Interferences from Endogenous Substances

Endogenous interferents were spiked directly into serum samples prior to adding red blood cells to make contrived blood samples, then spotted onto DBS cards (Table 20). To account for dilution of the serum with the interferent a paired (un-spiked) serum was diluted with a confirmed negative serum (blank) using the same dilution ratio. The spiked and un-spiked serum were then made into contrived blood samples and spotted onto DBS cards. All samples followed the recommended drying and overnight storage, samples were extracted the following day and results were compared to paired samples without interferents. For acceptance criteria of results, the FDA guidelines were used for quantitative and qualitative analysis *(3,5)*.

Table 21: Endogenous interferent spiking scheme

| **Sun Diagnostics Interferent (Catalog #)** | **Test Concentration** | **Interferent Concentration** | **Serum Relative Volume** | **Interferent Relative Volume** |
| --- | --- | --- | --- | --- |
| Triglycerides  (INT-01T) | 3000 mg/dL | >15,000 mg/dL (5X) | 4 parts | 1 part |
| Total Protein  (INT-01P) | 12 g/dL* | >20 g/dL (2X) | 4 parts | 3 parts |
| Bilirubin, conjugated  (INT-01B) | 20 mg/dL | >400 mg/dL (20X) | 19 parts | 1 part |
| Bilirubin, unconjugated  (INT-01BU) | 20 mg/dL | >400 mg/dL (20X) | 19 parts | 1 part |
| Biotin  (INT-09) | 3,510 ng/mL | 70,200 ng/mL (20X) | 19 parts | 1 part |
| *Assuming a serum concentration of 6 g/dL. | | | | |

## DBS Triglyceride Interference Study

When spiking samples with triglycerides at a concentration of 3000 mg/dL, DBS results were found to have a categorical agreement of 96.7%. The one sample that did not have categorical agreement had a baseline concentration of 0.179 U/mL which is within 4.0% of the DBS clinical cutoff (0.185 U/mL). Overall, the triglyceride spiked DBS samples had a mean bias of 4.5% compared to baseline results (Table 21). These results were acceptable under the FDA guidelines for the mean bias and categorical agreement *(3,5)*.

Table 22: DBS triglycerides interference results

| Sample | Baseline | | Triglyceride Interference (3000 mg/dL) | | | |
| --- | --- | --- | --- | --- | --- | --- |
|  | U/mL | Result | U/mL | Bias | Result | Agree/ Disagree |
| 1 | 0.0512 | N | 0.0411 | NA | N | A |
| 2 | 0.0557 | N | 0.0676 | NA | N | A |
| 3 | 0.0595 | N | 0.0483 | NA | N | A |
| 4 | 0.0709 | N | 0.0527 | NA | N | A |
| 5 | 0.0733 | N | 0.0825 | NA | N | A |
| 6 | 0.0747 | N | 0.0411 | NA | N | A |
| 7 | 0.0791 | N | 0.144 | NA | N | A |
| 8 | 0.0797 | N | 0.0731 | NA | N | A |
| 9 | 0.0809 | N | 0.0703 | NA | N | A |
| 10 | 0.0835 | N | 0.073 | NA | N | A |
| 11 | 0.136 | N | 0.121 | NA | N | A |
| 12 | 0.179 | N | 0.218 | NA | P | D |
| 13 | 0.21 | P | 0.228 | 8.6% | P | A |
| 14 | 0.296 | P | 0.380 | 28.4% | P | A |
| 15 | 0.314 | P | 0.328 | 4.5% | P | A |
| 16 | 0.389 | P | 0.386 | -0.8% | P | A |
| 17 | 0.459 | P | 0.392 | -14.6% | P | A |
| 18 | 0.461 | P | 0.363 | -21.3% | P | A |
| 19 | 0.732 | P | 0.786 | 7.4% | P | A |
| 20 | 0.768 | P | 0.797 | NA | P | A |
| 21 | 0.86 | P | 0.876 | 1.5% | P | A |
| 22 | 0.861 | P | 1.21 | 40.5% | P | A |
| 23 | 0.945 | P | 0.860 | -9.0% | P | A |
| 24 | 0.96 | P | 0.797 | -17.0% | P | A |
| 25 | 1.01 | P | 1.19 | 17.8% | P | A |
| 26 | 1.02 | P | 0.999 | -2.1% | P | A |
| 27 | 1.05 | P | 1.08 | 2.9% | P | A |
| 28 | 1.1 | P | 1.09 | -0.9% | P | A |
| 29 | 1.53 | P | 1.72 | 12.4% | P | A |
| 30 | 1.60 | P | 1.90 | 18.8% | P | A |
|  |  |  | Mean Bias: | 4.5% | Agreement: | 96.7% |

N – negative, P – positive, A – results agree, D – results disagree

## DBS Total Protein Interference Study

Following spiking of DBS samples to create additive protein concentrations of 12 g/dL, measurements indicated a total qualitative categorical agreement of 100% and mean bias of 17.7% when compared to baseline measurements (Table 22). These results were acceptable under the FDA criteria for the mean bias and categorical agreement *(3,5)*.

Table 23: DBS total protein interference results

| Sample | Baseline | | Protein Interference (12 g/dL) | | | |
| --- | --- | --- | --- | --- | --- | --- |
|  | U/mL | Result | U/mL | Bias | Result | Agree/ Disagree |
| 1 | 0.0532 | N | 0.0667 | NA | N | A |
| 2 | 0.0567 | N | 0.0834 | NA | N | A |
| 3 | 0.0572 | N | 0.0973 | NA | N | A |
| 4 | 0.0629 | N | 0.112 | NA | N | A |
| 5 | 0.0635 | N | 0.0995 | NA | N | A |
| 6 | 0.0675 | N | 0.144 | NA | N | A |
| 7 | 0.0678 | N | 0.0894 | NA | N | A |
| 8 | 0.0690 | N | 0.110 | NA | N | A |
| 9 | 0.0778 | N | 0.0618 | NA | N | A |
| 10 | 0.0818 | N | 0.0969 | NA | N | A |
| 11 | 0.130 | N | 0.159 | NA | N | A |
| 12 | 0.240 | P | 0.281 | 17.1% | P | A |
| 13 | 0.252 | P | 0.340 | 34.9% | P | A |
| 14 | 0.265 | P | 0.326 | 23.0% | P | A |
| 15 | 0.268 | P | 0.355 | 32.5% | P | A |
| 16 | 0.289 | P | 0.278 | -3.8% | P | A |
| 17 | 0.291 | P | 0.369 | 26.8% | P | A |
| 18 | 0.313 | P | 0.377 | 20.4% | P | A |
| 19 | 0.317 | P | 0.370 | 16.7% | P | A |
| 20 | 0.328 | P | 0.363 | 10.7% | P | A |
| 21 | 0.336 | P | 0.418 | 24.4% | P | A |
| 22 | 0.386 | P | 0.455 | 17.9% | P | A |
| 23 | 0.395 | P | 0.404 | 2.3% | P | A |
| 24 | 0.580 | P | 0.582 | 0.3% | P | A |
| 25 | 0.604 | P | 0.816 | 35.1% | P | A |
| 26 | 0.625 | P | 0.644 | 3.0% | P | A |
| 27 | 0.653 | P | 0.609 | -6.7% | P | A |
| 28 | 0.685 | P | 0.877 | 28.0% | P | A |
| 29 | 0.714 | P | 1.03 | 44.3% | P | A |
| 30 | 1.07 | P | 1.18 | 10.3% | P | A |
|  |  |  | Mean Bias: | 17.7% | Agreement: | 100.0% |

N – negative, P – positive, A – results agree, D – results disagree

## DBS Bilirubin Interference Studies

Spiking of conjugated bilirubin (up to 20 mg/dL) into DBS samples demonstrated a categorical agreement of 96.7% with baseline DBS samples with a mean bias of -2.8% (Table 23). The one sample that did not have categorical agreement had a baseline concentration of 0.0653 U/mL and a spiked concentration of 0.215 U/mL which is within 16.0% of the DBS clinical cutoff (0.185 U/mL). Spiking of DBS samples with unconjugated bilirubin demonstrated a categorical agreement of 100.0% with baseline results with a mean bias of -8.7% (Table 23). These results were acceptable under the FDA guidance for mean bias and categorical agreement *(3,5)*.

Table 24: DBS conjugated and unconjugated bilirubin interference results

| Sample | Baseline | | Conjugated Bilirubin Results | | | | Unconjugated Bilirubin Results | | | | |  |
| --- | --- | --- | --- | --- | --- | --- | --- | --- | --- | --- | --- | --- |
|  | U/mL | Result | U/mL | Bias | Result | Agree/ Disagree | | U/mL | Bias | Result | Agree/ | |
|  |  |  |  |  |  |  |  |  |  |  | Disagree | |
| 1 | 0.0653 | N | 0.215 | NA | P | D | | 0.140 | NA | N | A | |
| 2 | 0.0781 | N | 0.151 | NA | N | A | | 0.0941 | NA | N | A | |
| 3 | 0.0873 | N | 0.106 | NA | N | A | | 0.0939 | NA | N | A | |
| 4 | 0.0950 | N | 0.122 | NA | N | A | | 0.0880 | NA | N | A | |
| 5 | 0.101 | N | 0.134 | NA | N | A | | 0.0871 | NA | N | A | |
| 6 | 0.103 | N | 0.0707 | NA | N | A | | 0.0662 | NA | N | A | |
| 7 | 0.105 | N | 0.0886 | NA | N | A | | 0.0671 | NA | N | A | |
| 8 | 0.109 | N | 0.0901 | NA | N | A | | 0.0697 | NA | N | A | |
| 9 | 0.114 | N | 0.0788 | NA | N | A | | 0.0637 | NA | N | A | |
| 10 | 0.132 | N | 0.134 | NA | N | A | | 0.0798 | NA | N | A | |
| 11 | 0.283 | P | 0.248 | -12.4% | P | A | | 0.251 | -11.3% | P | A | |
| 12 | 0.326 | P | 0.343 | 5.2% | P | A | | 0.352 | 8.0% | P | A | |
| 13 | 0.429 | P | 0.430 | 0.2% | P | A | | 0.362 | -15.6% | P | A | |
| 14 | 0.449 | P | 0.412 | -8.2% | P | A | | 0.365 | -18.7% | P | A | |
| 15 | 0.453 | P | 0.431 | -4.9% | P | A | | 0.454 | 0.2% | P | A | |
| 16 | 0.470 | P | 0.426 | -9.4% | P | A | | 0.399 | -15.1% | P | A | |
| 17 | 0.472 | P | 0.509 | 7.8% | P | A | | 0.499 | 5.7% | P | A | |
| 18 | 0.490 | P | 0.521 | 6.3% | P | A | | 0.542 | 10.6% | P | A | |
| 19 | 0.495 | P | 0.444 | -10.3% | P | A | | 0.387 | -21.8% | P | A | |
| 20 | 0.558 | P | 0.594 | 6.5% | P | A | | 0.409 | -26.7% | P | A | |
| 21 | 1.11 | P | 1.02 | -8.1% | P | A | | 0.941 | -15.2% | P | A | |
| 22 | 1.13 | P | 1.05 | -7.1% | P | A | | 1.17 | 3.5% | P | A | |
| 23 | 1.15 | P | 1.10 | -4.3% | P | A | | 0.985 | -14.3% | P | A | |
| 24 | 1.19 | P | 1.24 | 4.2% | P | A | | 1.17 | -1.7% | P | A | |
| 25 | 1.32 | P | 1.32 | 0.0% | P | A | | 1.19 | -9.8% | P | A | |
| 26 | 1.35 | P | 1.31 | -3.0% | P | A | | 1.11 | -17.8% | P | A | |
| 27 | 1.42 | P | 1.33 | -6.3% | P | A | | 1.20 | -15.5% | P | A | |
| 28 | 1.47 | P | 1.35 | -8.2% | P | A | | 1.23 | -16.3% | P | A | |
| 29 | 1.59 | P | 1.42 | -10.7% | P | A | | 1.50 | -5.0% | P | A | |
| 30 | 2.10 | P | 2.26 | 7.6% | P | A | | 2.19 | 4.3% | P | A | |
|  |  |  | Mean Bias: | -2.8% | Agreement: | 96.7% | | Mean Bias: | -8.7% | Agreement: | 100.0% | |

N – negative, P – positive, A – results agree, D – results disagree

## Interferences from Biotin on DBS

Biotin interference was tested by spiking DBS samples to a biotin concentration of 3,510 ng/mL. A total categorical agreement of 96.6% with baseline DBS samples was observed (Table 24). The one sample that did not agree with baseline measurements had a measured baseline concentration (0.205 U/mL) within 11% of the DBS clinical cutoff (0.185 U/mL). These results indicate that DBS measurements in the presence of biotin levels up to 3,510 ng/mL are acceptable under the FDA guidance for the mean bias and categorical agreement *(3,5)*.

Table 25: DBS biotin interference results

| Sample | Baseline | | Biotin (3510ng/mL) | | | |
| --- | --- | --- | --- | --- | --- | --- |
|  | U/mL | Result | U/mL | Bias | Result | Agree/ Disagree |
| 1 | 0.112 | N | 0.123 | NA | N | A |
| 2 | 0.125 | N | 0.0852 | NA | N | A |
| 3 | 0.128 | N | 0.0859 | NA | N | A |
| 4 | 0.149 | N | 0.0766 | NA | N | A |
| 5 | 0.154 | N | 0.0958 | NA | N | A |
| 6 | 0.170 | N | 0.0929 | NA | N | A |
| 7 | 0.182 | N | 0.103 | NA | N | A |
| 8 | 0.205 | P | 0.101 | NA | N | D |
| 9 | 0.245 | P | 0.199 | -18.8% | P | A |
| 10 | 0.324 | P | 0.344 | 6.2% | P | A |
| 11 | 0.328 | P | 0.215 | -34.5% | P | A |
| 12 | 0.347 | P | 0.332 | -4.3% | P | A |
| 13 | 0.352 | P | 0.302 | -14.2% | P | A |
| 14 | 0.397 | P | 0.337 | -15.1% | P | A |
| 15 | 0.511 | P | 0.502 | -1.8% | P | A |
| 16 | 0.575 | P | 0.596 | 3.7% | P | A |
| 17 | 0.643 | P | 0.648 | 0.8% | P | A |
| 18 | 0.700 | P | 0.837 | 19.6% | P | A |
| 19 | 0.710 | P | 0.798 | 12.4% | P | A |
| 20 | 0.938 | P | 1.04 | 10.9% | P | A |
| 21 | 1.07 | P | 0.989 | NA | P | A |
| 22 | 1.16 | P | 1.33 | 14.7% | P | A |
| 23 | 1.17 | P | 1.36 | 16.2% | P | A |
| 24 | 1.26 | P | 1.46 | 15.9% | P | A |
| 25 | 1.30 | P | 1.39 | 6.9% | P | A |
| 26 | 1.34 | P | 1.49 | 11.2% | P | A |
| 27 | 1.37 | P | 1.64 | 19.7% | P | A |
| 28 | 4.21 | P | 4.73 | 12.4% | P | A |
| 29 | 7.63 | P | 7.93 | 3.9% | P | A |
|  |  |  | Mean Bias: | 3.3% | Agreement: | 96.6% |

N – negative, P – positive, A – results agree, D – results disagree

## Interferences from Pharmaceutical Substances on DBS Analysis

Serum samples were spiked with pharmaceutical interferents (from Cerilliant Analytical Reference Standards) and then red blood cells were added to make contrived blood samples that were spotted on DBS cards (Table 25). To account for dilution of the serum with the interferent a paired (un-spiked) serum was diluted with a confirmed negative serum (blank) using the same dilution ratio. The spiked and un-spiked serum were then made into contrived blood samples and spotted onto DBS cards. Samples dried for the recommended time of three hours prior to storage.

Table 26: Pharmaceutical substance interference spiking scheme

| **Cerilliant Interference Mix (Catalog #)** | **Pharmaceutical Substance** | **Test Concentration (µg/mL)** | **Interferent Concentration (µg/mL)** | **Serum Relative Volume** | **Interferent Relative Volume** |
| --- | --- | --- | --- | --- | --- |
| 1 (I-023) | (-)-Cotinine | 100 | 1000 | 9 parts | 1 part |
|  | (-)-Nicotine | 100 | 1000 |  |  |
|  | Acetaminophen | 100 | 1000 |  |  |
|  | Caffeine | 100 | 1000 |  |  |
|  | Ibuprofen | 100 | 1000 |  |  |
|  | Naproxen | 100 | 1000 |  |  |
|  | Phentermine | 10 | 100 |  |  |
|  | R,R(-)-Pseudoephedrine, List I | 10 | 100 |  |  |
| 2 (I-024) | Gabapentin | 10 | 100 | 9 parts | 1 part |
|  | Pregabalin | 10 | 100 |  |  |
|  | Salicylic acid | 10 | 100 |  |  |
|  | Valproic acid | 10 | 100 |  |  |
|  | Vigabatrin | 10 | 100 |  |  |

DBS samples spiked with Cerilliant mix 1 had a total categorical agreement of 96.6% with baseline DBS results (Table 26). One of the thirty Cerilliant mix 1 DBS samples was in disagreement with baseline; DBS results were less than 1% greater than the DBS clinical cutoff (0.186 versus the cutoff of 0.185 U/mL). Mean bias was 5.8% when compared to baseline results. Samples that were spiked with Cerilliant mix 2 had a total categorical agreement of 100.0% with baseline DBS results. Overall, Cerilliant mix 2DBS results had a mean bias -0.2% when compared to baseline DBS results (Table 27). These results indicate that DBS samples containing supra-physiological levels of tested drugs from Cerilliant mix 1 and 2 are acceptable under the FDA guidance for the mean bias and categorical agreement *(3,5)*. Cerilliant mix 1 and 2 DBS results were within the acceptance criteria provided by the FDA.

Table 27: DBS Cerilliant Mix 1 interference results

| Sample | Baseline | | Cerilliant Mix 1 | | | |
| --- | --- | --- | --- | --- | --- | --- |
|  | U/mL | Result | U/mL | Bias | Result | Agree/ Disagree |
| 1 | 0.0575 | N | 0.0771 | NA | N | A |
| 2 | 0.0580 | N | 0.117 | NA | N | A |
| 3 | 0.0646 | N | 0.0959 | NA | N | A |
| 4 | 0.0653 | N | 0.100 | NA | N | A |
| 5 | 0.0660 | N | 0.0769 | NA | N | A |
| 6 | 0.0772 | N | 0.0564 | NA | N | A |
| 7 | 0.0864 | N | 0.0869 | NA | N | A |
| 8 | 0.0910 | N | 0.0727 | NA | N | A |
| 9 | 0.104 | N | 0.0547 | NA | N | A |
| 10 | 0.171 | N | 0.186 | NA | P | D |
| 11 | 0.226 | P | 0.311 | 37.6% | P | A |
| 12 | 0.252 | P | 0.246 | -2.4% | P | A |
| 13 | 0.276 | P | 0.262 | -5.1% | P | A |
| 14 | 0.434 | P | 0.415 | -4.4% | P | A |
| 15 | 0.467 | P | 0.403 | -13.7% | P | A |
| 16 | 0.469 | P | 0.435 | -7.2% | P | A |
| 17 | 0.514 | P | 0.487 | -5.3% | P | A |
| 18 | 0.528 | P | 0.469 | -11.2% | P | A |
| 19 | 0.546 | P | 0.578 | 5.9% | P | A |
| 20 | 0.881 | P | 0.717 | -18.6% | P | A |
| 21 | 0.940 | P | 1.01 | 7.4% | P | A |
| 22 | 1.03 | P | 1.26 | 22.3% | P | A |
| 23 | 1.14 | P | 1.35 | 18.4% | P | A |
| 24 | 1.18 | P | 1.22 | 3.4% | P | A |
| 25 | 1.21 | P | 1.07 | -11.6% | P | A |
| 26 | 1.31 | P | 1.25 | -4.6% | P | A |
| 27 | 1.60 | P | 1.56 | -2.5% | P | A |
| 28 | 1.95 | P | 1.91 | -2.1% | P | A |
| 29 | 3.54 | P | 3.26 | -7.9% | P | A |
|  |  |  | Total Bias: | 5.8 | Total Agreement: | 96.6% |

N – negative, P – positive, A – results agree, D – results disagree

Table 28: DBS Cerilliant Mix 2 interference results

| Sample | Baseline | | Cerilliant Mix 2 | | | |
| --- | --- | --- | --- | --- | --- | --- |
|  | U/mL | Result | U/mL | Bias | Result | Agree/ Disagree |
| 1 | 0.0575 | N | 0.0638 | NA | N | A |
| 2 | 0.0580 | N | 0.0701 | NA | N | A |
| 3 | 0.0646 | N | 0.0735 | NA | N | A |
| 4 | 0.0653 | N | 0.0722 | NA | N | A |
| 5 | 0.0660 | N | 0.111 | NA | N | A |
| 6 | 0.0772 | N | 0.0459 | NA | N | A |
| 7 | 0.0864 | N | 0.0512 | NA | N | A |
| 8 | 0.0910 | N | 0.0523 | NA | N | A |
| 9 | 0.104 | N | 0.0591 | NA | N | A |
| 10 | 0.171 | N | 0.177 | NA | N | A |
| 11 | 0.226 | P | 0.238 | 5.3% | P | A |
| 12 | 0.252 | P | 0.238 | -5.6% | P | A |
| 13 | 0.276 | P | 0.254 | -8.0% | P | A |
| 14 | 0.434 | P | 0.460 | 6.0% | P | A |
| 15 | 0.467 | P | 0.420 | -10.1% | P | A |
| 16 | 0.469 | P | 0.482 | 2.8% | P | A |
| 17 | 0.514 | P | 0.461 | -10.3% | P | A |
| 18 | 0.528 | P | 0.494 | -6.4% | P | A |
| 19 | 0.546 | P | 0.524 | -4.0% | P | A |
| 20 | 0.881 | P | 0.866 | -1.7% | P | A |
| 21 | 0.940 | P | 1.12 | 19.1% | P | A |
| 22 | 1.03 | P | 1.10 | 6.8% | P | A |
| 23 | 1.14 | P | 1.31 | 14.9% | P | A |
| 24 | 1.18 | P | 1.17 | -0.8% | P | A |
| 25 | 1.21 | P | 1.11 | -8.3% | P | A |
| 26 | 1.31 | P | 1.52 | 16.0% | P | A |
| 27 | 1.60 | P | 1.71 | 6.9% | P | A |
| 28 | 1.95 | P | 2.11 | 8.2% | P | A |
| 29 | 3.54 | P | 3.61 | 2.0% | P | A |
|  |  |  | Total Bias: | -0.2% | Total Agreement: | 100.0% |

N – negative, P – positive, A – results agree, D – results disagree

# References

1. Clinical and Laboratory Standards Institute. EP17-A2 Evaluation of Detection Capability for Clinical Laboratory Measurement Procedures; Approved Guideline. Second. 2012.

2. Food and Drug Administration. Bioanalytical Method Validation Guidance for Industry. Food Drug Adm. 2018;

3. U.S. Food & Drug Administration. EUA Authorized Serology Test Performance. Vol. 2, FDA. 2020. p. 1–14.

4. U.S. Food & Drug Administration. Home Specimen Collection Serology Template for Fingerstick Dried Blood Spot [Internet]. 2020. Available from: https://www.fda.gov/medical-devices/coronavirus-disease-2019-covid-19-emergency-use-authorizations-medical-devices/vitro-diagnostics-euas

5. International Safe Transit Association. Temperature Test for Transport Packaging; ISTA 7 Series Developemt Test Procedure. 2013.

# Abbreviations

CLSI Clinical and Laboratory Standards Institute

CV Coefficient of variance

DBS Dried Blood Spot

DF Degrees of Freedom

EUA Emergency Use Authorization

FDA Food and Drug Administration

MS Mean of Square

n Number of replicates

N Negative

NA (or N/A) Not Applicable

NPA Negative Predictive Agreement

NTP No test performed

NPV Negative Predictive Value

P Positive

PPA Positive Predictive Agreement

PPV Positive Predictive Value

QC Quality control

SD Standard Deviation

SS Sum of Squares

U/mL Units per milliliter
